# Coastal restoration evaluated using dominant habitat characteristics and associated fish communities

**DOI:** 10.1371/journal.pone.0240623

**Published:** 2020-10-22

**Authors:** Kailee Schulz, Philip W. Stevens, Jeffrey E. Hill, Alexis A. Trotter, Jared L. Ritch, Quenton M. Tuckett, Joshua T. Patterson

**Affiliations:** 1 Program in Fisheries and Aquatic Sciences, School of Forest Resources and Conservation, University of Florida, Gainesville, Florida, United States of America; 2 Fish and Wildlife Research Institute, Florida Fish and Wildlife Conservation Commission, St. Petersburg, Florida, United States of America; 3 Tropical Aquaculture Laboratory, University of Florida, Ruskin, Florida, United States of America; 4 Center for Conservation, The Florida Aquarium, Apollo Beach, Florida, United States of America; Swedish University of Agricultural Science, SWEDEN

## Abstract

Increasing coastal populations and urban development have led to the loss of estuarine habitats for fish and wildlife. Specifically, a decline in complexity and heterogeneity of tidal marshes and creeks is thought to negatively impact fish communities by altering the function of nursery grounds, including predator refuge and prey resources. To offset these impacts, numerous agencies are restoring degraded habitats while also creating new ones where habitat has been lost. To improve understanding of what contributes to a successful restoration, six quarterly sampling events using two gear types to collect small- and large-bodied fishes were conducted to compare the fish community structure and habitat characteristics at three natural, three restored, and three impacted (i.e. ditched) areas along the coast of Tampa Bay, Florida. Overall, impacted sites had significantly lower small-bodied and juvenile fish diversity than natural and restored areas, while restored sites harbored a greater number of fish species than impacted sites for both large- and small-bodied fish. Habitat features such as shoreline slope differentiated impacted and restored from natural areas. Although we did not find a direct correlation, habitat heterogeneity likely played a role in structuring fish communities. These findings provide guidance for future coastal restoration or modification of existing projects. Specifically, the habitat mosaic approach of creating a geographically compact network of heterogenous habitat characteristics is likely to support fish diversity, while decreasing shoreline slope in a greater amount of area within coastal wetland restorations would more closely mimic natural areas.

## Introduction

Coastal wetlands, namely saltmarshes and mangroves, support a rich assemblage of animals [1]. Many fishes are inextricably linked to coastal wetland habitats [2, 3], which are some of the most productive aquatic habitats globally [4]. An important aspect of the sub- and inter-tidal areas of coastal wetlands is habitat heterogeneity, which provides multiple habitat types and features. Habitat heterogeneity can increase species richness and diversity by providing a range of habitat features within near-shore ecosystems [5, 6]. Diverse habitat features provide structure and ultimately protection, food resources, spawning substrates, and nursery grounds for multiple species [1, 7, 8]. Near-shore ecosystems are characterized by a diversity of habitats (e.g. seagrass beds, mangrove forests, and oyster reefs [9, 10]), which are associated with high fish diversity and richness [11–15]. Diversity improves the resilience of living systems and is essential for sustaining valuable ecosystem services and marine natural resources [16–18]. Despite recognition of these benefits, waterfront development has caused a direct reduction in the area of coastal wetlands [5, 19, 20], affecting the fish assemblages that are dependent on them [14, 21].

Globally, residential structures on shorelines are expected to more than double in density from 2000 to 2060, when coasts are projected to support 534 residences per km^2^ [22]. In the same period, an additional 43.9 million people are expected to almost triple the population of the United States now living directly on a coast [22, 23]. Coastal development and population growth negatively affect estuarine habitat quality; development increases impermeable surfaces, fills in wetlands, alters water quality, and affects freshwater flow patterns [24, 25]. Physical impacts to estuaries are significant and ongoing, often necessitating restoration to improve ecosystem function. Restoration agencies often focus on large-scale restoration efforts [26], restoring or creating multiple habitat features with the goal of increasing the complexity and heterogeneity of nearshore environments [27–30].

Tampa Bay is Florida’s largest open-water estuary [31], with a coastal shoreline that naturally includes mangroves and salt marshes [32]. However, an increasing population and construction of deep-water ports have resulted in significant physical modifications [33]. This development led to the excavation or filling of approximately half of emergent coastal wetlands, including a reduction in mangrove and salt marsh shorelines and seagrass coverage [32, 34]. The result was reduced habitat heterogeneity and a reduction in spatially complex features [5, 27]. Due to physical modifications to meet the demands of an increasing population, Tampa Bay has become a focal region for restoration efforts [32, 35, 36]. However, despite a nearly 50-year history of ecological restoration in Tampa Bay, little is known about the effects of restoration, especially large-scale efforts, on fish communities. Early studies examining multiple restoration sites found similar fish populations to those of natural sites, both in species composition and abundance, within five years of construction [37, 38]. This provided justification for resource managers to move forward with designing and implementing additional large-scale restorations in Tampa Bay [39]. Outside these early studies, limited research has been conducted to understand the habitat features that support diverse and species-rich communities, especially at restored locations.

From a practical standpoint, fish community structure and diversity can be used to assess the condition of estuaries and the success of restoration projects [40]. The goal of the present study was to determine how restored habitats compare to impacted sites and more natural areas in terms of 1) habitat heterogeneity and 2) fish community structure. The premise was that heterogeneous habitats provide more niches and opportunities to exploit environmental resources, thus increasing species diversity [41]. To address this goal, we compared habitat characteristics and fish communities in coastal wetlands at restored sites to those at sites that were natural or impacted. We expected impacted sites to have low habitat heterogeneity relative to natural sites while restored sites, which are designed to have high habitat heterogeneity, should support fish communities similar in diversity to natural sites. To test these ideas, a stratified random sampling design using two gear types was employed across nine locations along the shorelines of Tampa Bay.

## Materials and methods

### Study area

**Fig 1:**
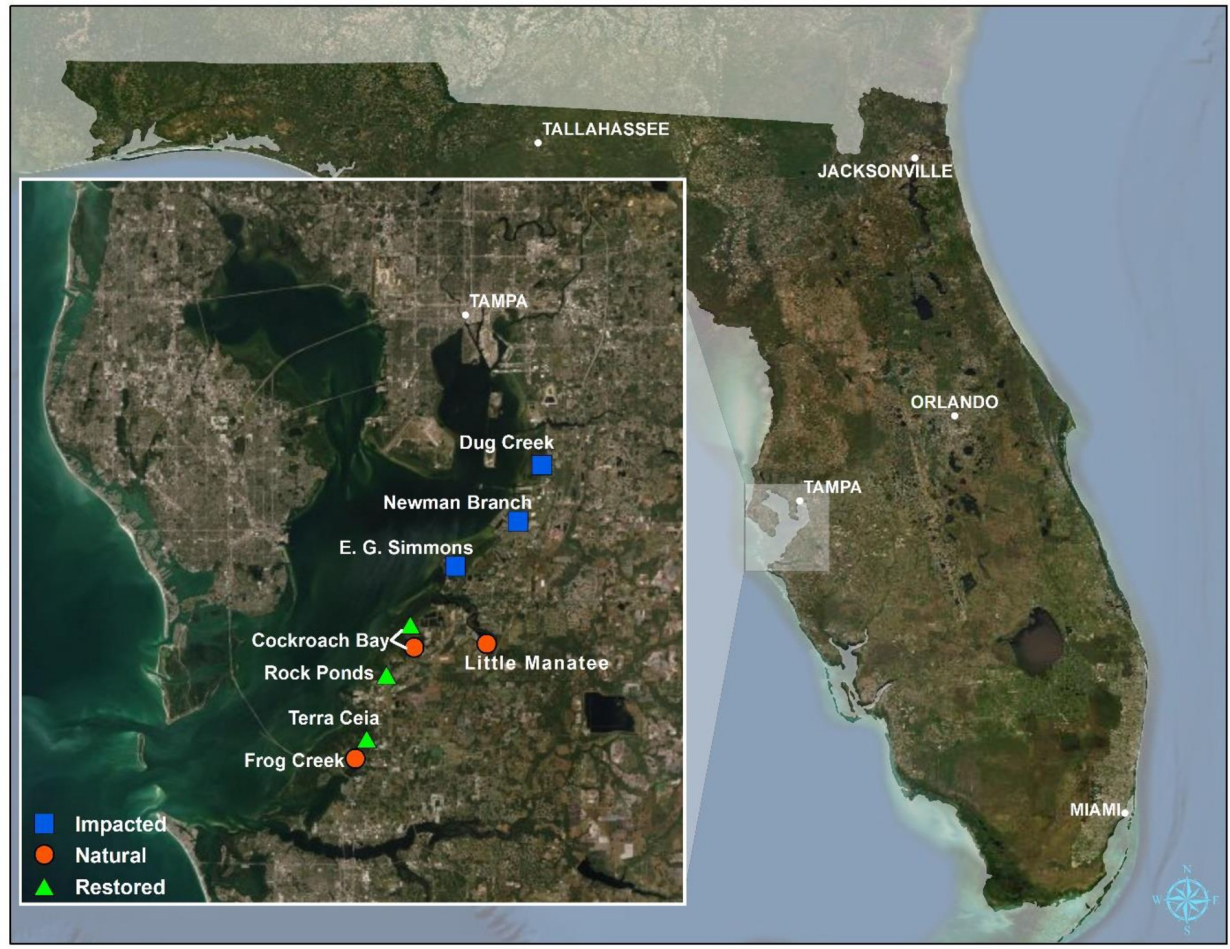
Satellite image of Florida with an inset of Tampa Bay, showing the location of nine study sites. Colors and shapes correspond to the designated site type. Sites are abbreviated as follows: Cockroach Bay Natural (CBN), Cockroach Bay Restored (CBR), Dug Creek (DC), E.G. Simmons (EG), Frog Creek (FC), Little Manatee (LM), Newman Branch (NB), Rock Ponds (RP), Terra Ceia (TC). Map images were sources from the State of Florida via ESRI.

The study area was located on the eastern shore of Tampa Bay and included three natural, three restored, and three impacted sampling areas (Fig 1). Sites were selected based on accessibility, proximity to adjacent sites, and published works describing fish communities within Tampa Bay [2, 36, 42, 43]. A restored site was defined as an area that has been physically and biologically modified to re-establish or create habitat that supports estuarine aquatic communities. An impacted site was classified as a historically dredged canal or ditch that received minimal subsequent modification [44, 45]. A natural site was distinguished as an area with minimal physical and biological alteration to aquatic habitat that would provide suitable reference for comparison to restored and impacted areas. Such sites are difficult to identify [46] and were located by examining historical aerial and satellite imagery on Google Earth (Google, Mountain View, CA, USA). A site was classified as natural if no shoreline or connectivity changes were evident since the 1950s, when shoreline development increased in Tampa Bay [32, 39]. Due to limitations in viable sites and patterns of development and restoration, impacted locations are primarily to the north of other site types, which could potentially result in spatial autocorrelation. Environmental variables including salinity were rigorously tracked to help account for this limitation in experimental design. At first mention of each site below, geographic coordinates (dd°mm’ss.ss”) are provided for the central point of the sampled area.

Restored sites included Rock Ponds Ecosystem Restoration (N27°39’03.31” W82°32’01.51”), Terra Ceia Ecosystem Restoration (N27°36’04.01” W82°33’06.03”), and Cockroach Bay Restoration (N27°41’31.40” W82°30’30.97”). Rock Ponds Ecosystem Restoration Project (RP) was coordinated by the Southwest Florida Water Management District (SWFWMD) and involved physically and biologically engineering an aquatic ecosystem from agricultural fields [47]. Approximately 422 hectares of various coastal habitats, including 107 hectares of upland and 127 hectares of estuarine and freshwater habitats, were created. To improve the hydrology of the area, tidal connections were added, increasing variation in depth and substrate components [47]. The restoration was expansive and thus the sampling area was limited to the western restoration section, which was completed in 2012 [47]. Terra Ceia Ecosystem Restoration Project (TC) was coordinated by SWFWMD and includes a mosaic of 294 hectares of coastal uplands and 47 hectares of freshwater and estuarine habitats [48]. The project, completed in 2010, involved restoring estuarine habitats historically modified by dredge-and-fill activities. The Cockroach Bay Restoration Project (CBR) was completed in 1991 and was coordinated by Hillsborough County Environmental Lands and Protection Program. Over the course of 20 years, 202 hectares of wetlands, uplands, and coastal habitats were restored. Restoration activities included a pond, palustrine marsh restoration, and construction of a braided tidal creek. The study area included all three habitat types.

The impacted sites were Dug Creek (N27°49’35.30” W82°23’20.63”), Newman Branch (N27°46’56.10” W82°24’38.01”), and dredged canals at E.G. Simmons Park (N27°44’27.69” W82°28’02.23”). Dug Creek (DC) was historically channelized into a 1.2 m-deep ditch to provide drainage for 327 hectares of uplands, a common practice in Tampa Bay in the 1940s and 1950s [49]. The sampling area included a 1,037 m dredged channel that flows into Tampa Bay and a parallel 831 m channel. These channels were connected tidally by two smaller ditches. Newman Branch (NB) was comprised of a 1,500 m dredged channel created for recreational boating access to Tampa Bay and a ditch constructed for upland drainage. E.G. Simmons Park (EG) was developed in the 1960s from 104 hectares of native mangrove. The interior 81 hectares of mangroves were protected from habitat modification. The sampling area included channels dredged to facilitate recreation and access to Tampa Bay and excluded the protected, natural mangrove area.

Natural sites comprised areas within Little Manatee River (N27°41’01.74” W82°26’37.81”), Cockroach Bay (N27°40’43.80” W82°30’17.32”), and Frog Creek (N27°35’05.25” W82°33’46.77”). Little Manatee River (LM) flows approximately 64 km through southern Hillsborough County and into Tampa Bay. The study area was limited to approximately 5.3 km of shoreline and connected creeks within a small embayment (Hayes Bayou) of LM. The Cockroach Bay natural site (CBN) is an embayment just south of CBR. Approximately 4.71 km of shoreline was sampled and included one tidally connected creek. The sampling area within Frog Creek (FC) excluded the northern, inland portion, concentrating on an estimated 1.15 km of narrow creeks and island habitats. For more information on the ecology and history of FC see [42] and [50].

### Sampling site selection

Using ArcGIS (10.3, Esri, Redlands, CA), a standard WGS grid system (1 minute of latitude = 1 nautical mile) was fragmented into a grid representing 1/10^th^ of a coordinate second, or polygons that are roughly 18 meters of latitude by 16 meters of longitude. This grid, referred to as the “minigrid,” was then overlaid on aerial imagery of the site locations. Minigrid squares that intersected accessible shoreline were incorporated into a site’s sampling universe, and all other minigrid squares were discarded. The size of some sites required that the minigrid be divided into two to three smaller grids to preserve even stratification of the randomly selected sample locations. The Python language and ArcGIS ModelBuilder were used to create a tool that randomly selected a total number of minigrid squares from within a site’s sampling universe. Each selected minigrid square was then assigned one sampling gear type. At each site, 12 minigrid squares were selected for 9.1-m seine sampling (9 primary samples and 3 alternates) and 6 minigrid squares were selected for 40-m seine sampling (3 primary samples and 3 alternates) for each sampling event (see net description and sampling frequency below). Alternates were only used when one of the primary samples were not suitable for a seine pull (e.g., site was inaccessible). Thus, the maximum number of seine pulls conducted for any site on a given day was 12. Duplicate selections were not allowed within a sampling event. If a selected minigrid square contained multiple shorelines, a random number generator was used to select the shoreline to be sampled.

### Field sampling

To collect animals from a natural setting we obtained a field permit (SAL-17-1952-SR) from Florida Fish and Wildlife Conservation Commission. From January 2018 through June 2019, fishes were collected quarterly (n = 6) at each of the nine sites for a total of 54 sampling events (see locations in S1 Fig). Sampling during each quarter was completed within six weeks and all sampling occurred during daylight hours. A nylon beach seine (9.14 m ×1.65 m with 3-mm mesh, hereafter referred to as 9-m seine) and a larger seine (39.6 m × 2.4 m, with 25.4-mm mesh, hereafter referred to as 40-m seine) were used for up to 9 and 3 pulls per sampling event, respectively. On five occasions, adverse field conditions prevented crews from completing all 12 seine pulls on a given sampling day. To standardize 9-m seine pulls for area sampled, pull width was set at 8 m, accounting for net curve, and an 8-m pull length was established. This created an 8 m × 8 m seined area. Pull length was adjusted when depth exceeded 1.4 m, whereupon distance from seine bag to shore was measured. The 40-m seine was deployed from the bow of the boat in a half circle away from the shoreline. Sampling depth was limited by net height (1.3 m). Sampling area for each pull was calculated assuming distance to shore equaled the length of one end of the net to the beginning of the bag (35 m). Collected fishes were identified to species, except fishes of the family Atherinopsidae (New World silversides) and small specimens (<50 mm total length, TL) of the family Gerridae (mojarras), using field guides [51, 52]. All animals were counted and released, except a subset of common snook (*Centropomus undecimalis*) less than 200 mm TL, which were retained for a separate study [53] or specimens with ambiguous field identifications that were retained for laboratory verification.

Principal habitat characteristics at the location of each seine pull were visually estimated across the sampled area to the nearest whole percentage and recorded by the same observer throughout the study. Recorded habitat characteristics included the type and percent coverage of bottom vegetation, substrate composition (percent sand, mud, rubble, etc.) and composition of shoreline vegetation. These parameters were estimated using standard Florida Fish and Wildlife Conservation Commission Fisheries Independent Monitoring protocols [54]. Water temperature (°C), pH, and salinity (ppt) were measured (YSI Pro 1030, YSI Inc., Yellow Springs, OH, USA) mid-column at each sampling location. Shoreline slope was determined by measuring water depth at the bag and at the shore for each seine pull. Water transparency was determined with a transparency tube (FREY scientific, Nashua, NH, USA). Air temperature (°C) was recorded using a mercury thermometer. Location coordinates were verified with a handheld GPS (Garmin eTrex 10, Olathe, KS, USA). Tide position was documented after data collection from NOAA tide charts in relation to the time of each seine pull.

### Data analysis

All analyses were performed in R Program version 3.4.1. Packages specific to each analysis are denoted in parentheses. To analyze habitat characteristics and fish community data, a combination of GLMs and ordinations were performed. A principal components analysis (PCA) was performed to identify important habitat characteristics among the nine sites (Stats package version 3.4.1). Habitat characteristics included water conditions (salinity, temperature, transparency), percent substrate composition (mud, sand, shell, oysters, rubble) and shoreline vegetation. Shoreline species identified at more than 10 seine pulls were included in the PCA, which removed rare shoreline vegetation. After removing rare species, analyzed shoreline species included red mangrove (*Rhizophora mangle*), black mangrove (*Avicennia germinans*), white mangrove (*Laguncularia racemose*), black needlerush (*Juncus roemerianus*), cordgrass (*Spartina* sp.), sawgrass (*Cladium jamaicense*), and Brazilian pepper (*Schinus terebinthifolius*).

Shoreline slope, water temperature, salinity, pH, and transparency were independently compared between site types (natural, restored, impacted) using generalized linear models (GLM). Habitat heterogeneity was assessed using descriptive statistics and an index of diversity, depending upon whether heterogeneity can be calculated for each sampling location. Descriptive statistics for each site, including the coefficient of variation (CV), were calculated for salinity, shoreline slope, total percent shoreline vegetation coverage, number of different shoreline plant species present, and the number of different substrate types present at each site. For a subset of these variables, substrate and shoreline vegetation, we calculated Simpson’s diversity (s) at each sampling location. Unlike the PCA, diversity calculations also included rare species. A gamma GLM model was used to find differences in Simpson’s diversity for substrate and vegetation among sites (package lme4 version 1.1–21). Least means analysis was used post hoc to identify site differences.

Prior to data analysis, the fish capture data (counts) in the 9-m and 40-m seine hauls fish density data were averaged per quarter to reduce the number of zero-catch data. The number of fish captured in the 9-m and 40-m seine hauls were analyzed separately due to differences in mesh size and net length and, therefore, catch. In addition to fish density, the following indices were calculated for each net pull: species richness (S), the Shannon-Wiener Diversity index (h), and Evenness (E) [55]. These diversity indices, along with total fish counts, were subsequently analyzed using a nested generalized linear mixed effects model (GLMM; package lme4 version 1.1–21). For all models, the categorical variable site was included as a nested random variable within the categorical fixed variable site type (natural, restored, or impacted) The underlying distribution of each dependent variable was determined by visual inspection of the data and AIC scores. If a significant main effect of site type was detected, least means analysis was used post hoc to assess pairwise differences among sites and site types (package emmeans version 1.3.4). The fish capture data was fit to the negative binomial distribution (in comparison to a Poisson and zero-inflated negative binomial), which accounted for over dispersion in the density data (package glmmTMB version 0.2.3). Sampling area was included as an offset. Diversity indices were fit to the Gaussian distribution. A series of linear models was used to assess correlations between fish diversity or species richness and the heterogeneity calculations, including shoreline and substrate heterogeneity and CV of slope and salinity (Stats package version 3.4.1). The remaining three CV calculations were omitted to avoid redundancy with the shoreline and substrate heterogeneity.

Differences in fish assemblages among sites were visualized using nonmetric multidimensional scaling (NMDS) (Vegan package version 2.5–4). Bray-Curtis similarities were first calculated based on joint occurrence and abundance of taxa [56]. Square-root transformed density data was analyzed for each of the six quarters at each of the nine sites (total of 54). Resulting axes were compared with fish species and taxonomic groups to identify patterns in species composition among sites.

## Results

**Fig 2:**
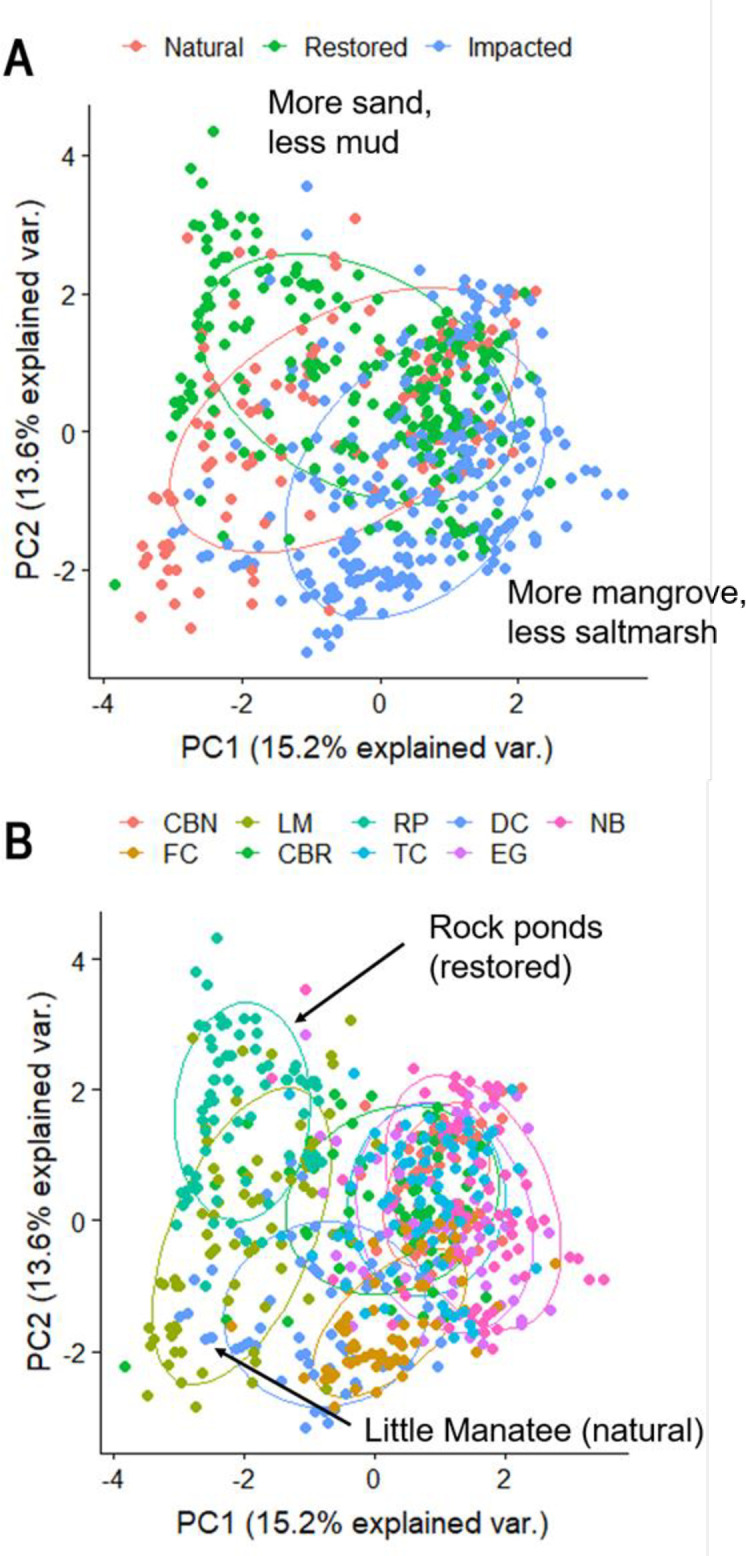
Principal component analysis (PCA) of dominant habitat characteristics throughout the sampling universe. Each point represents an individual seine pull at the denoted site type and site. A) PCA representing site types, B) PCA representing the nine sites. Sites are abbreviated as follows: CBN = Cockroach Bay natural, FC = Frog Creek, LM = Little Manatee River, CBR = Cockroach Bay restored, RP = Rock ponds, TC = Terra Ceia, DC = Dug Creek, EG = E.G. Simmons Park, NB = Newman Branch.

**Fig 3:**
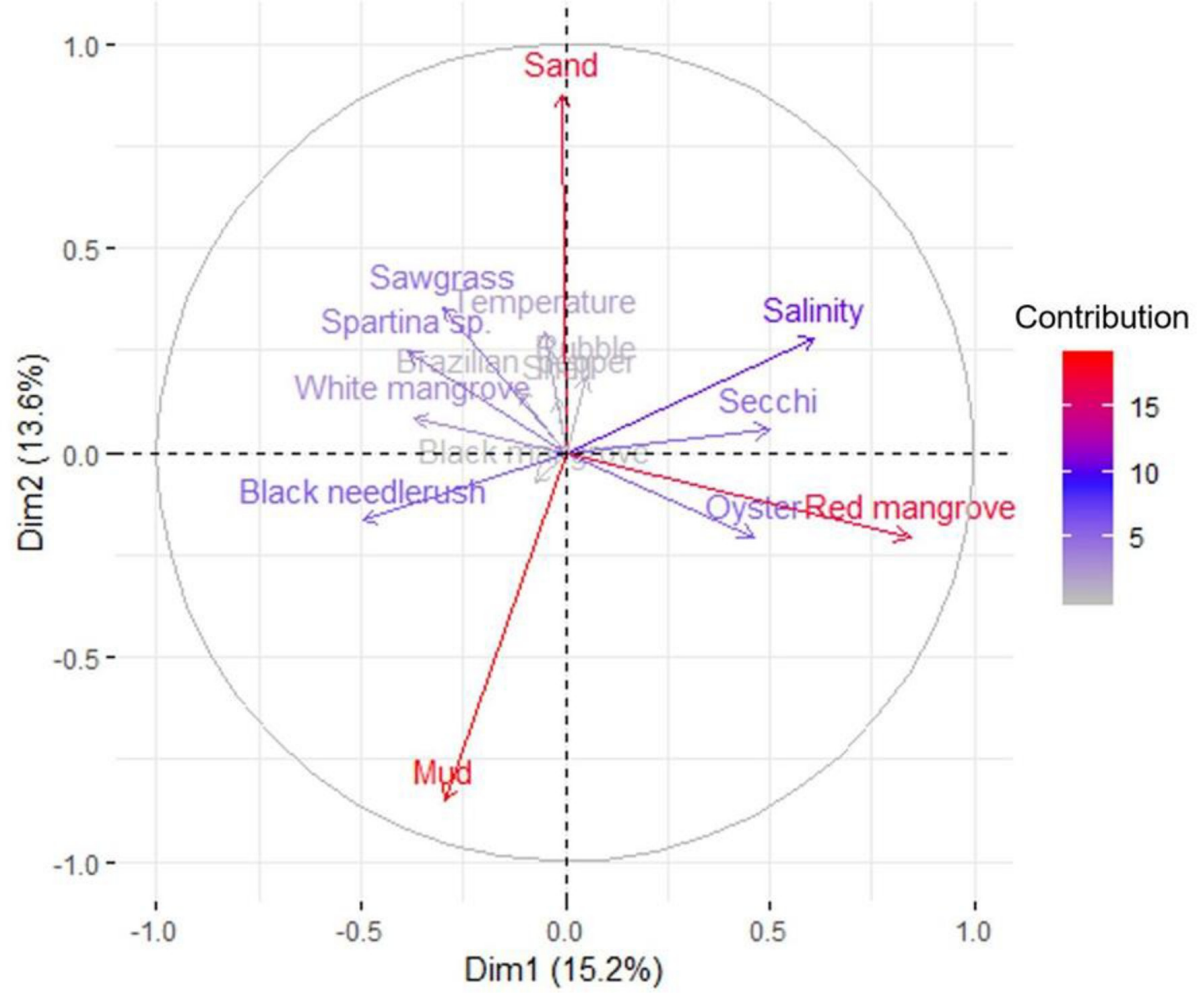
Habitat characteristic vectors for the PCAs displayed in Fig 2. The legend corresponds to the contribution of each variable to the PCA.

The PCA represented percent coverage of individual habitat characteristics, vegetation and substrate, as well as water variables, sand revealed strong associations for sites and particularly site types (Figs 2 and 3). The first and second PCA axes explained 14.9% and 13.4% of variation, respectively. The first component axis was positively associated with red mangrove shoreline coverage and negatively associated with black needlerush shoreline coverage. The second axis was positively associated with sand substrate and negatively associated with mud substrate. Thus, the first axis largely described a habitat gradient related to shoreline vegetation, and the second axis described a gradient of substrates among samples. The high degree of site overlap in Fig 2B is due to the occurrence of red mangrove covered shorelines, especially at impacted locations. Two sites that separate from the cluster, LM and RP, load negatively on the PC1 axis because of a correlation with the occurrence of emergent marsh grasses including sawgrass and Spartina species (Fig 3). Little Manatee River (LM) is a natural site with a mud substrate and a dense covering of black needlerush. A variety of shoreline vegetation, including emergent marsh grasses such as sawgrass and *Spartina* sp., was present at restored site RP, which also had a bare, sand substrate throughout. An oyster-dominated substrate and red mangrove coverage was associated with impacted sites but was also strongly present at Frog Creek (FC), a natural location.

**Fig 4:**
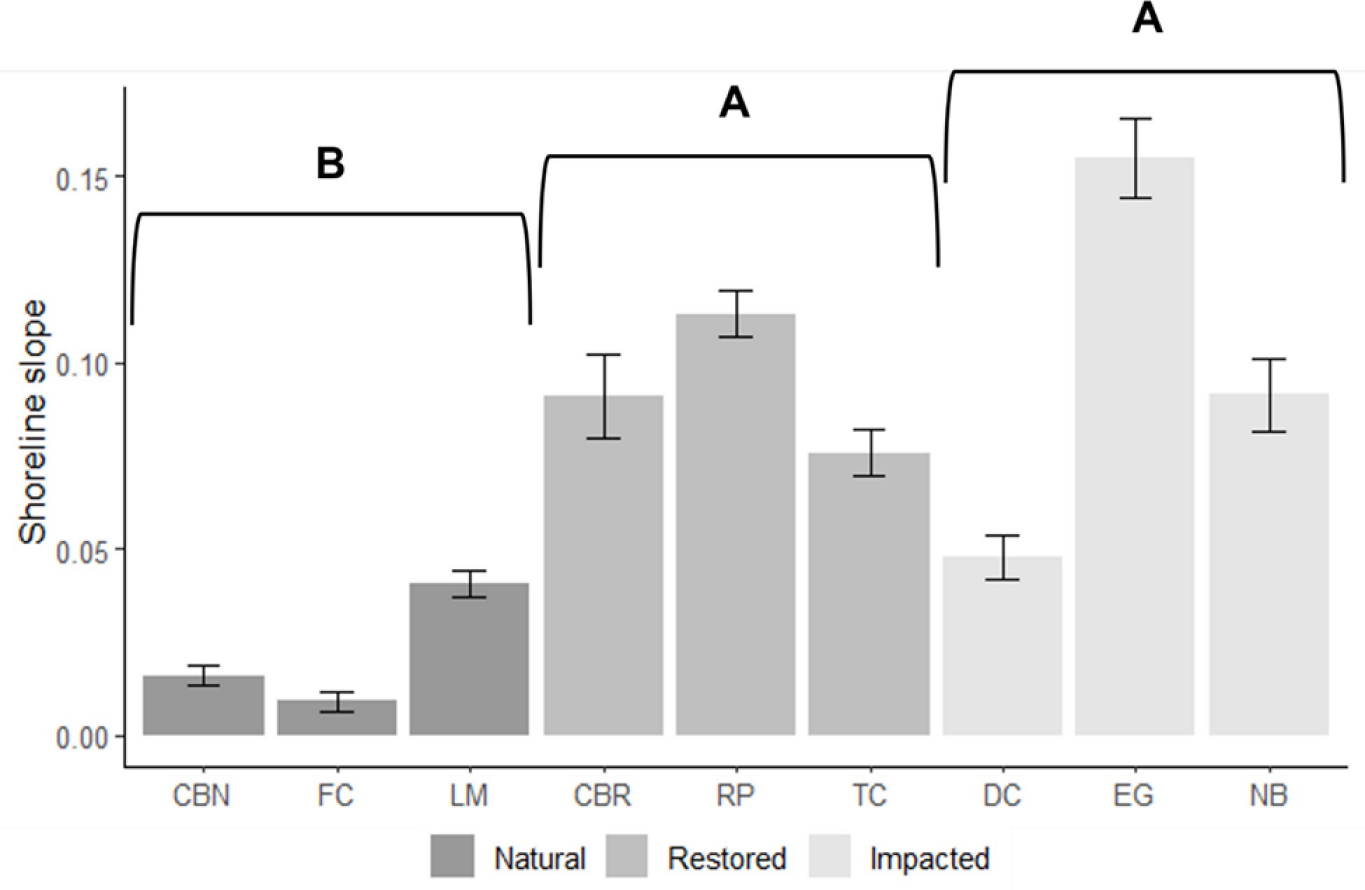
The mean (±SE) shoreline slope between the nine individual sites. The legend corresponds to site type. Sites are abbreviated as follows: CBN = Cockroach Bay natural, FC = Frog Creek, LM = Little Manatee River, CBR = Cockroach Bay restored, RP = Rock ponds, TC = Terra Ceia, DC = Dug Creek, EG = E.G. Simmons Park, NB = Newman Branch. Letters denote significant statistical differences among site types at α = 0.05.

The coefficient of variation in number of shoreline plant species present was similar among the three site types (Table 1), with FC, a natural site, exhibiting the lowest variation. Restored locations had the lowest variation in substrate types because of the sand-dominated habitats; more oyster or shell presence was often found at impacted and natural locations. Water temperature (*X*^*2*^_*2*_ = 1.577, *p* = 0.455), transparency (*X*^*2*^_*2*_ = 4.684, *p* = 0.096), salinity (*X*^*2*^_*2*_ = 2.569, *p* = 0.277), and pH (*X*^*2*^_*2*_ = 1.273, *p* = 0.529) were similar among the three site types. Slope varied among site types (*X*^*2*^_*2*_ = 9.273, *p* = 0.010; Fig 4), with shorelines at restored and impacted areas having a steeper slope than natural sites.

**Table 1:** The coefficient of variation (CV) of five habitat characteristics across site types and sampling areas.

Site	Slope	Salinity (ppt)	# Shoreline species	Total plant coverage	# Substrate types
**Natural**	**150.6**	**61.7**	**30.7**	**2.8**	**30.8**
CBN	135.7	15.9	39.9	0	24.3
FC	244.3	77.9	19.3	1.8	39.9
LM	71.8	91.4	33.9	6.5	28.1
**Restored**	**72.3**	**15.6**	**35.5**	**12.1**	**21.8**
CBR	104.9	15.2	38.3	7.8	22.6
RP	47.9	23.2	30.7	26.6	20.6
TC	64.2	8.3	37.5	1.9	22.1
**Impacted**	**84.3**	**28.6**	**37**	**8.1**	**34.9**
DC	104.3	51.9	33.1	13.6	39.2
EG	57.7	17.9	40.5	4.7	28.3
NB	90.9	15.9	37.5	5.9	37.1

The CV of each characteristic for a site type is in bold.

**Fig 5:**
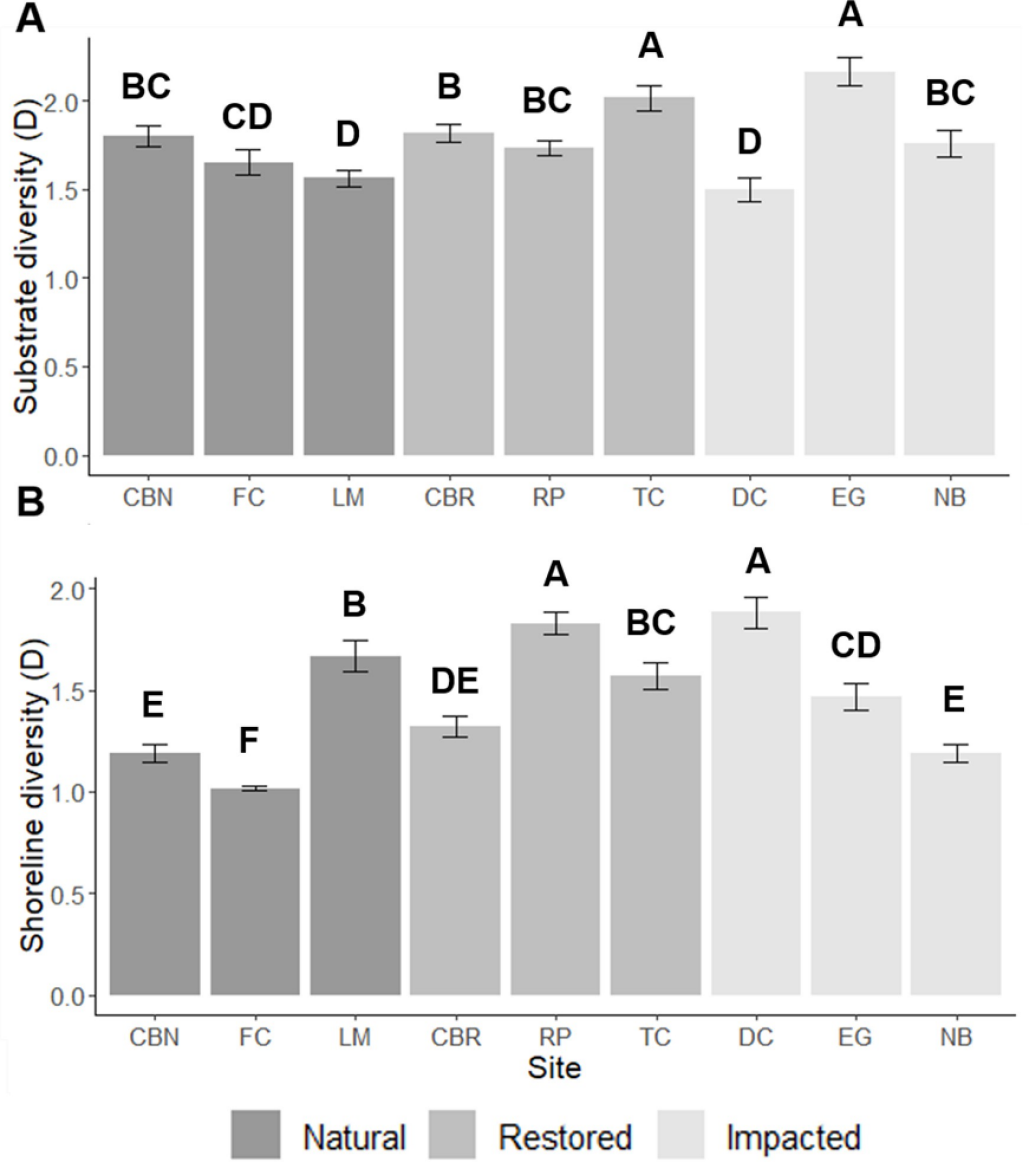
Mean Simpson’s diversity of substrates (±SE) (A) and number of shoreline vegetation types (B) across all sampling events at the nine sites. The legend corresponds to site type. Sites are abbreviated as follows: CBN = Cockroach Bay natural, FC = Frog Creek, LM = Little Manatee River, CBR = Cockroach Bay restored, RP = Rock ponds, TC = Terra Ceia, DC = Dug Creek, EG = E.G. Simmons Park, NB = Newman Branch. Letters denote significant statistical differences among sites at α = 0.05.

Shoreline vegetation and substrate diversity (surrogates for heterogeneity) varied among sites (shoreline vegetation: *X*^*2*^_*8*,*627*_ = 252.09, *p* ≤ 0.001; substrate: *X*^*2*^_*8*,*628*_ *=* 83.252, *p* ≤ 0.001; Fig 5); however, no pattern among site types emerged. Restored site, RP, and impacted site, DC, had high shoreline heterogeneity. A variety of marsh grass species was present at RP, which increased the diversity throughout the sample area. At DC, all three mangrove species and small patches of sawgrass created a heterogenous shoreline. Natural sites, CBN and FC, and impacted site, NB, had low shoreline heterogeneity, with shorelines dominated by red mangrove. Restored site, TC, and impacted site, EG, had the highest substrate heterogeneity, while natural site, LM, and impacted site, DC, had the lowest.

**Fig 6:**
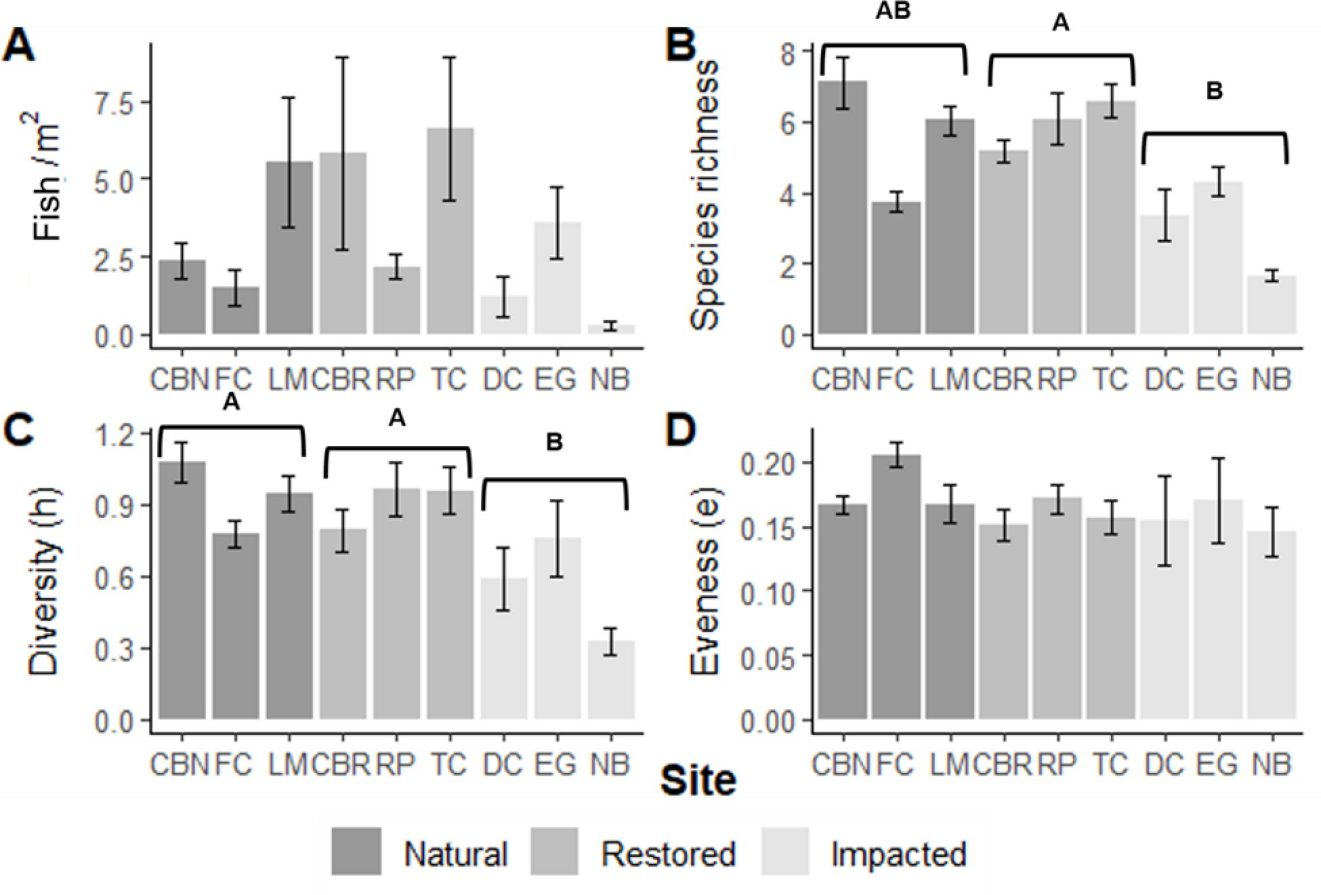
Fish community indices among the nine sites pooled over the entire sampling period for 9-meter seine pulls. The legend corresponds to site type. A) the mean (±SE) total fish density per seined m², B) species richness, C) Shannon-Wiener diversity (h), and D) and evenness. Letters denote significant statistical differences among site types at α = 0.05.

**Fig 7:**
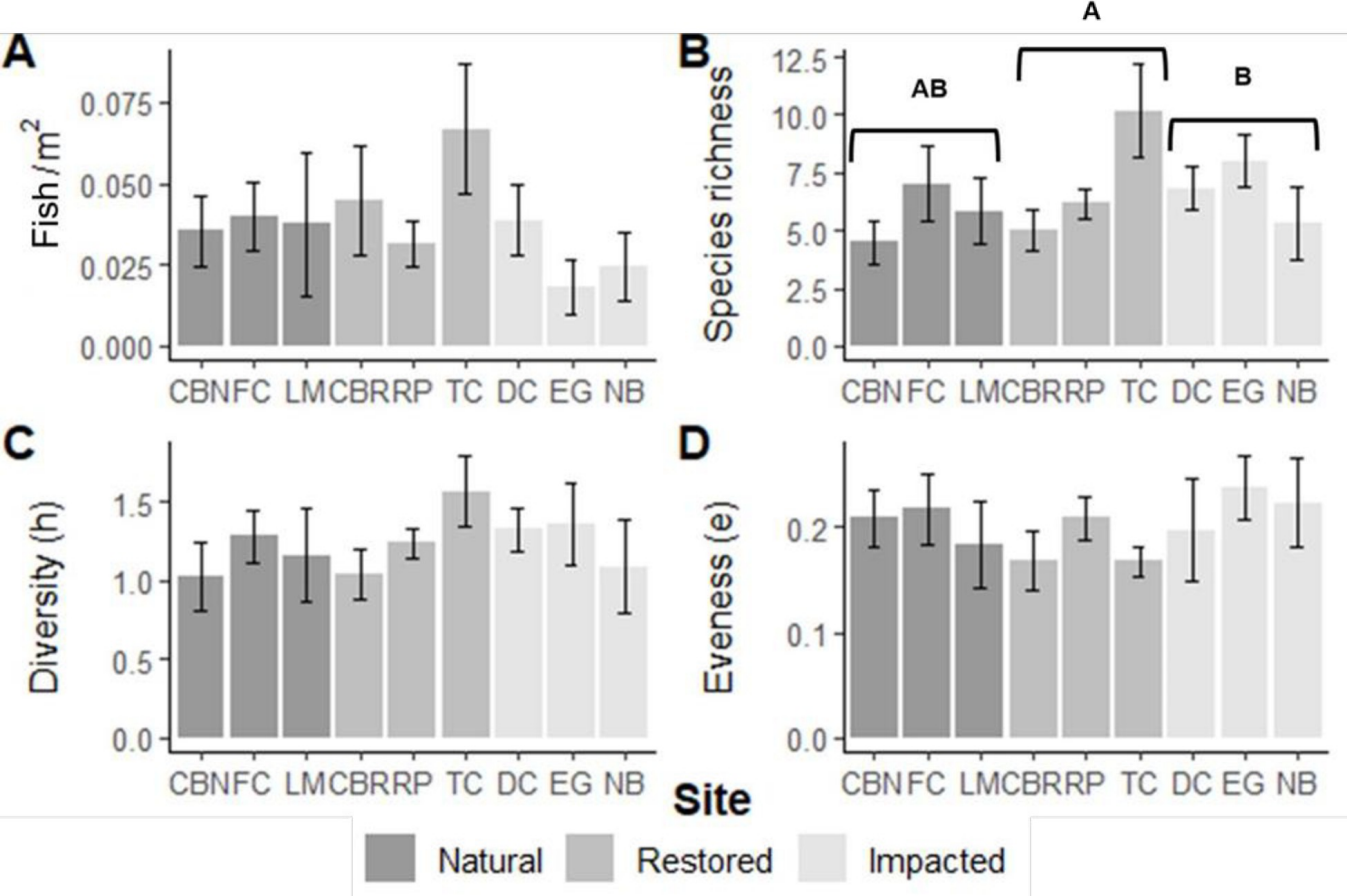
Fish community indices among the nine sites pooled over the entire sampling period for 40-meter seine pulls. The legend corresponds to site type. A) the mean (±SE) total fish density per seined m², B) species richness, C) Shannon-Wiener diversity (h), D) and evenness. Letters denote significant statistical differences among site types at α = 0.05.

During six quarters of sampling, 99,852 fishes were caught in 482 9-m seine hauls. Captured fishes included 67 species and 31 families (S1 Table). In comparison, 2,311 fishes were caught in 156 40-m seine hauls, including 53 species across 28 families. Density was significantly different among site types for 9-m and 40-m seine hauls (9-m: *X*^*2*^_*2*,*49*_ = 5602.5, *p <* 0.001; 40-m: *X*^*2*^_*2*,*49*_ = 2061.3, *p* < 0.001; Figs 6A and 7A). In the fine mesh 9-m seine, bay anchovy (*Anchoa mitchilli*, n = 67,325) and silverside species (Atherinopsidae sp.; n = 12,744), both common prey items of piscivorous fish, represented 80.18% of the total catch. Sportfish (i.e. fish with strict bag limits in Florida, USA; n = 1,097) were 1.10% of total fish abundance, and non-native fish accounted for less than 0.15% (n = 137). In total, 25 species were captured in <1% of the 9-m seine hauls and a total of 46 species were captured in <5% of seine hauls. Over a third of the fish captured (37.08%) in the 40-m seine were from the family Gerreidae (n = 857). Compared to the 9-m gear, sportfish (n = 402) and non-native fish (n = 62) represented a larger proportion of the total catch in the 40-m seine (17.31% and 2.68%, respectively). Five species were unique to the 40-m seine pulls, including tarpon (*Megalops atlanticus*) and great barracuda (*Sphyraena barracuda*).

Species richness differed among site types (9-m: *X*^*2*^_*2*_ = 8.172, *p* = 0.017; 40-m: *X*^*2*^_*2*_ = 8.833, *p* = 0.012; Figs 6B and 7B), with higher species richness at restored sites compared to impacted locations for both gear types. Several species were unique to one site type. Black drum (*Pogomias cromis*) was exclusive to all three restored areas and gulf flounder (*Paralichthys albiguttata*) was found at two restoration sites, CBR and TC. The crested goby (*Lophogobius cyprinoides*) was only captured at two impacted sites, EG and NB. Diversity differed among site types for the 9-m seine (*X*^*2*^_*2*_ = 9.881, *p* = 0.007; Fig 6C) but not for the 40-m seine (*X*^*2*^_*2*_ = 3.854, *p* = 0.146; Fig 7C). In general, 9-m seine hauls at impacted sites were less diverse than those at natural and restored sites. Evenness was not different among site types for both gears. Site-based linear models did not reveal an association between species diversity or richness and any heterogeneity measurements.

**Fig 8:**
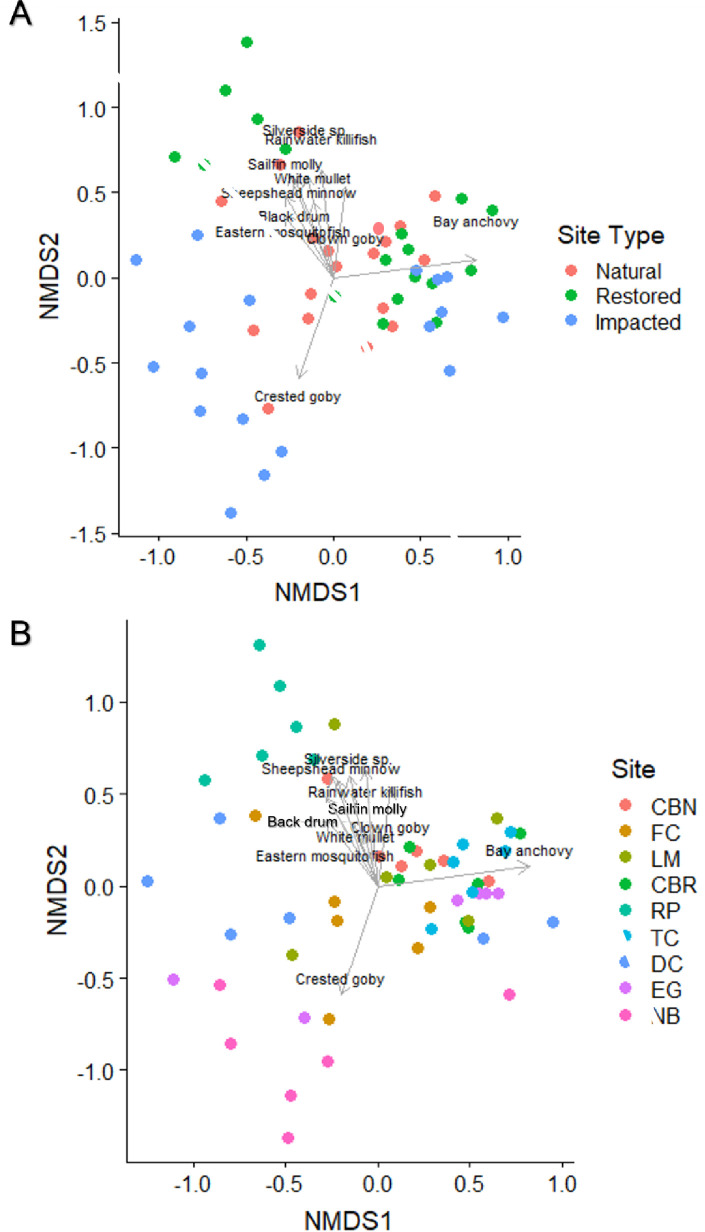
Non-metric multidimensional scaling ordination of sites in species space for 9-m seine hauls. Species are plotted in space based on their scores for each axis. Stress = 0.134 A) Quarterly samples are color coordinated by site type. B) Quarterly samples are color coordinated by site.

**Fig 9:**
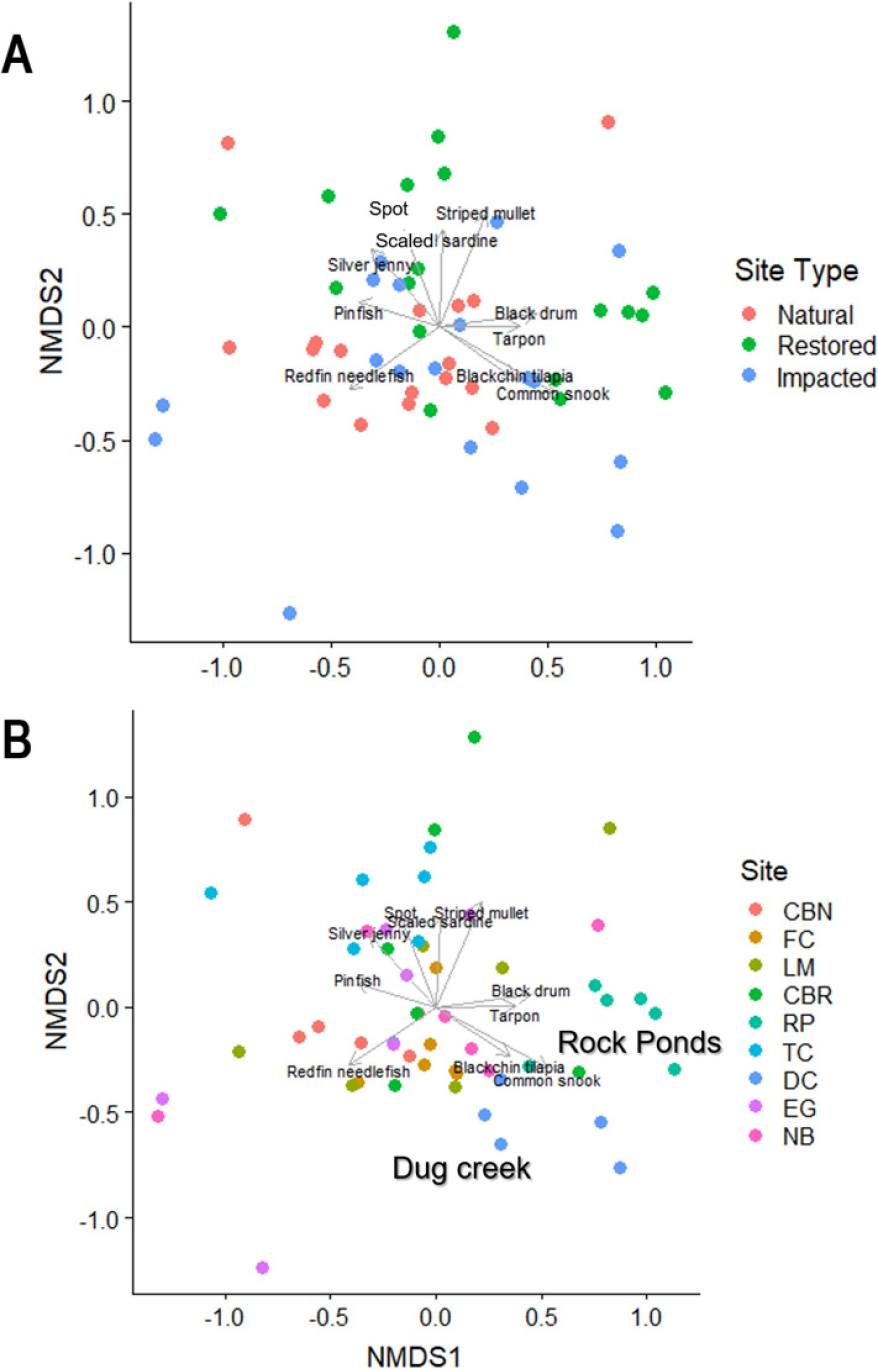
Non-metric multidimensional scaling ordination of sites in species space for 40-m seine hauls. Species are plotted in space based on their scores for each axis. Stress = 0.153 A) Quarterly samples are color coordinated by site type. B) Quarterly samples are color coordinated by site.

According to the NMDS of fish assemblages, averaged by sampling quarter for 9-m (Fig 8; stress = 0.134) and 40-m (Fig 9; stress = 0.153) seine hauls, a few species such as the crested goby were highly associated with specific sites; however, most were more broadly distributed across the nine sites (see S1 Data for loadings, sites, and species). Due to the high diversity of species caught in both gear types, the number of species vectors included on the NMDS plot was reduced for clarity at significance levels of *p* ≤ 0.01 for Fig 8 and *p* ≤ 0.02 for Fig 9.

## Discussion

One goal of physical restoration is to promote habitat complexity and heterogeneity, which is hypothesized to increase niche space, leading to greater species richness [8, 12]. Restoration efforts can include creation of habitat mosaics, where multiple habitat types are created within a single restoration [47]. This type of restoration should increase niche space and ultimately species richness. Our first objective was to determine whether habitat heterogeneity (i.e., substrate and vegetation diversity) varied among the nine sites and the three site types. In general, habitat heterogeneity was site specific; both substrate and shoreline vegetation heterogeneity differed among the nine sites. However, we did not find a clear difference/pattern between the three site types. Our second objective was to examine fish richness and diversity. Small-bodied fish species richness and diversity at restored sites were indistinguishable from natural sites, and higher compared to impacted sites, indicating that coastal shoreline restoration leads to robust fish communities similar to those found along natural shorelines. The results were similar with large-bodied fish, comparable species richness at restored and natural sites, which was higher than impacted sites. Restored sites are providing environmental and habitat conditions similar to natural sites, one of which is suitable shoreline vegetation for a variety of species [47], this is supported by the PCA. Ultimately, these results support the widely reported finding that many small fishes, including juvenile sportfish, utilize shoreline vegetation [24, 43, 54], making this habitat feature an important target for restoration.

Shoreline vegetation such as mangrove and marsh grass are important habitat mosaic components in Tampa Bay, but these habitats may fundamentally differ in their formation and maintenance and ultimately complexity and function [57]. Our results suggest that habitat heterogeneity was site specific. In particular, the newest restoration site, RP, had high shoreline vegetation diversity, composed of both salt marsh and white mangrove. This high level of shoreline heterogeneity may have been influenced by the age of the restoration. Tampa Bay environmental agencies focus on marsh grass restoration, allowing for mangroves to naturally recruit to the area [47], which suggests that in time RP will shift to a mangrove-dominated system like older sites CBR and TC. Restoration engineers plan for mangrove succession by planting saltmarsh to stabilize sediments, expecting mangrove species to eventually dominate, which is more cost effective than planting young mangroves which may be unable to quickly stabilize sediments [58].

Mangrove and marsh grass species occupy the intertidal zone of Florida, with periodic freeze events limiting mangrove expansion [59–62]. However, during the last three decades of relatively mild winters, mangroves have expanded into higher latitudes [63], generally outcompeting native marsh grasses following colonization [64, 65]. In natural habitats of Tampa Bay, mangroves are overtaking the remaining marsh grass covered coastlines [66]. Results of this study support this, as mangrove species covered most of the sampled shorelines including natural sites CBN and FC, impacted areas, and older restoration sites CBR and TC. However, a red mangrove dominated shoreline was not evident at Rock Ponds, the most recent restoration project. While many of these sites still had variation in vegetation cover along their shorelines, it was primarily a combination of three mangrove species. Both mangrove and marsh grasses increase the complexity of shorelines [67] but are associated with different physical characteristics and fish assemblages [1].

Shoreline slope and substrate characteristics are often directly manipulated during restoration projects in Tampa Bay. Substrate plays an important role in fish diversity and species richness [68, 69]. Features that add rugosity and complexity (i.e. oyster and seagrass species and artificial reefs) are important for many small-bodied fishes and sportfish throughout ontogeny [15, 70–74]. Large-scale restoration efforts often include the addition of rock features (e.g., artificial reefs composed of limestone at RP), but do not actively incorporate seagrass or oyster reefs [47]. Small quantities of seagrass were present at RP and CBR and oyster species were present on the mangrove roots at TC suggesting that the exclusion of these features in a mosaic still allows for natural accruement, but not in a substantial quantity. Slope is also an important component of habitat mosaics and has been associated with variation in fish communities [2, 75]. For example, shallow near-shore habitats likely provide increased refuge for small prey fish with steeper shorelines reflecting the abrupt, dredged banks of impacted areas, providing increased habitat for large, predatory species. Impacted and restored areas often exhibited lower variation in shoreline slope. While the two older restorations exhibited shallower slopes than RP, the most recent restoration, it is unclear whether slope will decline at restoration areas with time and sediment deposition. Substrate diversity did not vary among site types; however, restored and natural sites often exhibited more bare sand and mud in near-shore environments. Designing restorations with increased shoreline slope heterogeneity is likely to support more diverse fish communities, especially as shoreline vegetation diversity is altered by mangrove intrusion. Ultimately, however, if the goal of restoration is to mimic natural conditions, resource managers should emphasize restoration designs that emphasize shallow shoreline slope.

Our results indicate that the three impacted sites harbor less diverse small-bodied (i.e., fish captured in the 9-m seine) fish communities than natural and restored areas. These degraded areas also have lower species richness of both small and large-bodied fish communities than restored locations. However, there is variation in diversity indices and density across sites, with no specific site type having a consistently higher density, species richness, or diversity. According to the NMDS, fish assemblages did not exhibit strong associations with site type but did show some association with site. For example, RP was positive along NMDS1 and was separated from the other sites based on associations with tarpon, common snook, eastern mosquitofish (*Gambusia holbrooki*), and sailfin molly (*Poecilia latipinna*). At RP, high habitat heterogeneity and a shoreline mosaic of marsh grass and young mangroves resulting from recent restoration activity likely influenced the differences in fish communities.

Evidence from many studies suggests that habitat complexity and heterogeneity facilitate higher fish diversity, richness, and density [7, 8, 76, 77]. While habitat complexity, particularly rugosity and variation in growth forms, are positively associated with fish species richness [78], we were unable to separate the effects of habitat heterogeneity from complexity. Still, many of the sample locations also contained complex habitats (i.e., inundated physical structure providing surface area and protected spaces) such as mangroves, emergent grasses, or hard substrate components. Ultimately, we did not find an association between our indices of habitat heterogeneity and fish species richness and diversity. This result could be due to more important local factors overriding the influence of heterogeneity, the spatial scale of the analysis, or how heterogeneity was measured. Still, site-specific patterns, such as the unique assemblage at restored site RP and the high richness and diversity at restored site TC, reveal some interesting associations between habitat components and the fish community.

Restored site TC had comparatively high mean fish density, species richness, and diversity. The sampling area was comprised of two zones, a direct embayment of Tampa Bay and an inland area tidally connected via a culvert. The inland portion had a rubble dominated substrate, which was unique to the study. TC also had consistently high salinity due to its proximity to open water and lack of freshwater connections. This contrasted with RP, DC, and FC, sites with lower average salinity. The rubble substrate of TC, as well as the presence of oysters, was highly heterogeneous. The rubble added rugosity to the substrate and shoreline habitat, which was missing from other sampling areas. In environments from coral reefs to freshwater lakes, rugosity has been found to increase fish diversity, richness, and abundance by providing increased refuge for small fish species [30, 79]. These characteristics may have increased available niche space [6], potentially promoting the high species richness found at the site.

High species diversity and richness at natural site CBN was observed with the 9-m seine, indicating more small-bodied species along the shorelines. Slopes at CBN were very shallow, which may have promoted an abundance of small-bodied fish species because of the decreased risk of predation [75, 80]. Sections of CBN were also covered in shoal grass (*Halodule wrightii*), a common Tampa Bay seagrass, which increases the habitat complexity and provides refuge and a food source for many species [9]. Therefore, despite the homogenous shoreline vegetation, dominated by red mangrove, CBN may have a diverse fish assemblage due a high variation in substrate, specifically interchanges of submerged features (seagrass and oyster) and sections of shallow mud and sand shorelines. In comparison to CBN, natural site FC and impacted sites EG and DC exhibited low species richness and diversity in the 9-m seine but relatively high values for these metrics in 40-m seine hauls. Steeper slopes at EG and DC could have allowed for greater diversity and richness of large-bodied species. While FC had shallow sloping shorelines, deep pools present throughout the site likely provided viable habitat for large-bodied fish species. Low overall fish density and diversity at NB implies that factors at this site affected habitat suitability. The steep shoreline slope and homogeneity of red mangrove vegetation at NB, may have decreased the diversity juvenile and small-bodied fish species, impacting the availability of prey items for large-bodied predatory fish. NB was also the only site with a seawall adjacent to the sampling area, which has been shown to alter species assemblages and reduce fish abundance [81]. It is interesting to note that DC and FC, the northernmost and southernmost sites, had similar fish assemblages, suggesting that location within the Tampa Bay estuary may not be a primary factor in shaping fish assemblages.

The hypothesis that impacted shorelines have negative effects on fish diversity was supported by our data (i.e., diversity was significantly lower at impacted sites compared to restored and natural sites in the 9-m seine). Results also indicate that ecological restoration can lead to small- and large-bodied fish species diversity metrics similar to those in natural areas, suggesting that restoration has been successful by this metric. It is also interesting to note that neither overall fish abundance for either gear type or diversity of fishes captured in the 40-m seine were different among site types. This is likely because, with the exception of shoreline slope, habitat characteristics varied among individual sites but not among site-types. Despite the noted habitat variability, each of the nine sites had much in common with the others and thus differences among site types in all fish community metrics should likely not be expected to occur.

Causes of local diversity are difficult to identify because of conflation between heterogeneity and complexity. It was hypothesized that habitat heterogeneity would lead to increased fish species richness and diversity, a hypothesis that has widespread support [5, 6, 12]. Variation in habitat characteristics, slope and salinity, were highest at natural sites, and restored sites had the highest variation in plant coverage. These two site types also exhibited the highest fish species richness (small- and large-bodied) and diversity (small-bodied), but there was not a direct connection between heterogeneity and fish species richness. The interaction of numerous variables precludes recommendations on specific habitat characteristics (i.e., shoreline vegetation species, benthic substrate types) that support specific fishes. It is possible to draw broader conclusions. For example, the monotypic stands of red mangrove and steep shoreline slopes that characterized impacted habitats likely support high abundance only of specific species that thrive in such habitats (e.g. crested goby), with decreased species richness and diversity as related outcomes. Results suggest that managers can enhance local fish diversity by creating habitat mosaics. Mosaics consider a multitude of biotic and abiotic conditions, which again make it difficult to discern conditions that support community diversity and success. Therefore, creating a true habitat mosaic that includes a diversity of shoreline, substrate, and slope may enhance fish communities at restoration sites. A substrate mosaic with distinct patches of rubble, sand, and mud along with a combination of shallow and sloping shorelines may promote increased community diversity. Future research might evaluate relationships between fish community metrics and specific habitat or environmental characteristics on a smaller scale. Restoration efforts are critical to the future health and success of estuarine ecosystems and continued research will be necessary to optimize habitat functionality.

## Supporting information

S1 Fig Randomly selected sampling locations within each of the nine study sites throughout the study duration. Map images were sources from the State of Florida via ESRI.
(DOCX)

S1 Table Complete list of fish species captured during the study, quantified by number of individuals in both 9-m and 40-m seines. (DOCX)

S2 Table A list of the commercially and/or recreationally important sportfish captured during the study. (DOCX)

S1 Data Complete set of data and metadata underlying all reported findings. (XLSX)
