# Supplementary material for: Coastal restoration evaluated using dominant habitat characteristics and associated fish communities
Source: PLoS One. 2020 Oct 22;15(10):e0240623. doi: 10.1371/journal.pone.0240623 (PMC7580894; doi:10.1371/journal.pone.0240623)

**S1. Locations of seine pulls within each of the nine study sites in Tampa Bay.**

S1A. Natural Site Cockroach Bay (CBN)


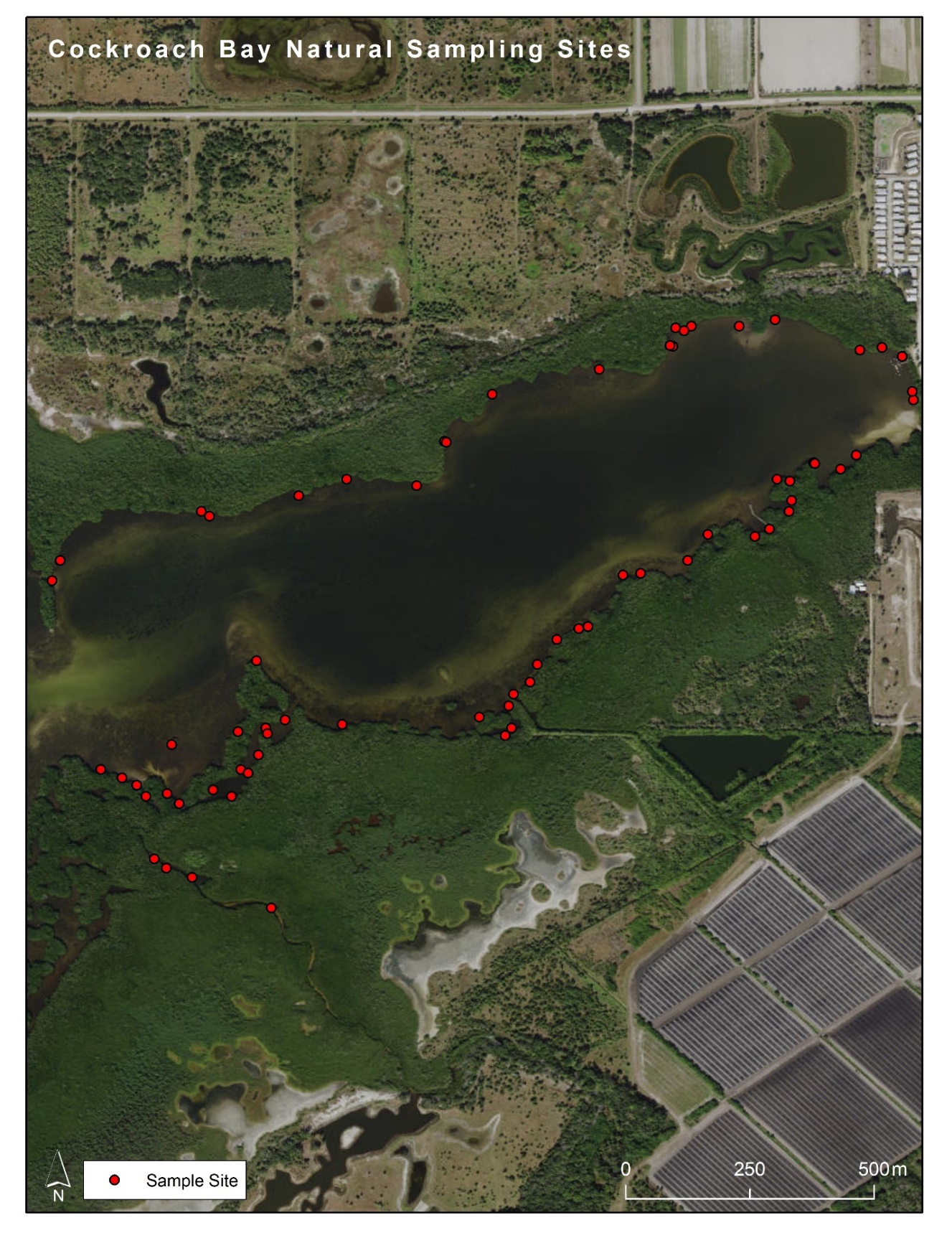


S1B. Natural Site Frog Creek (FC)


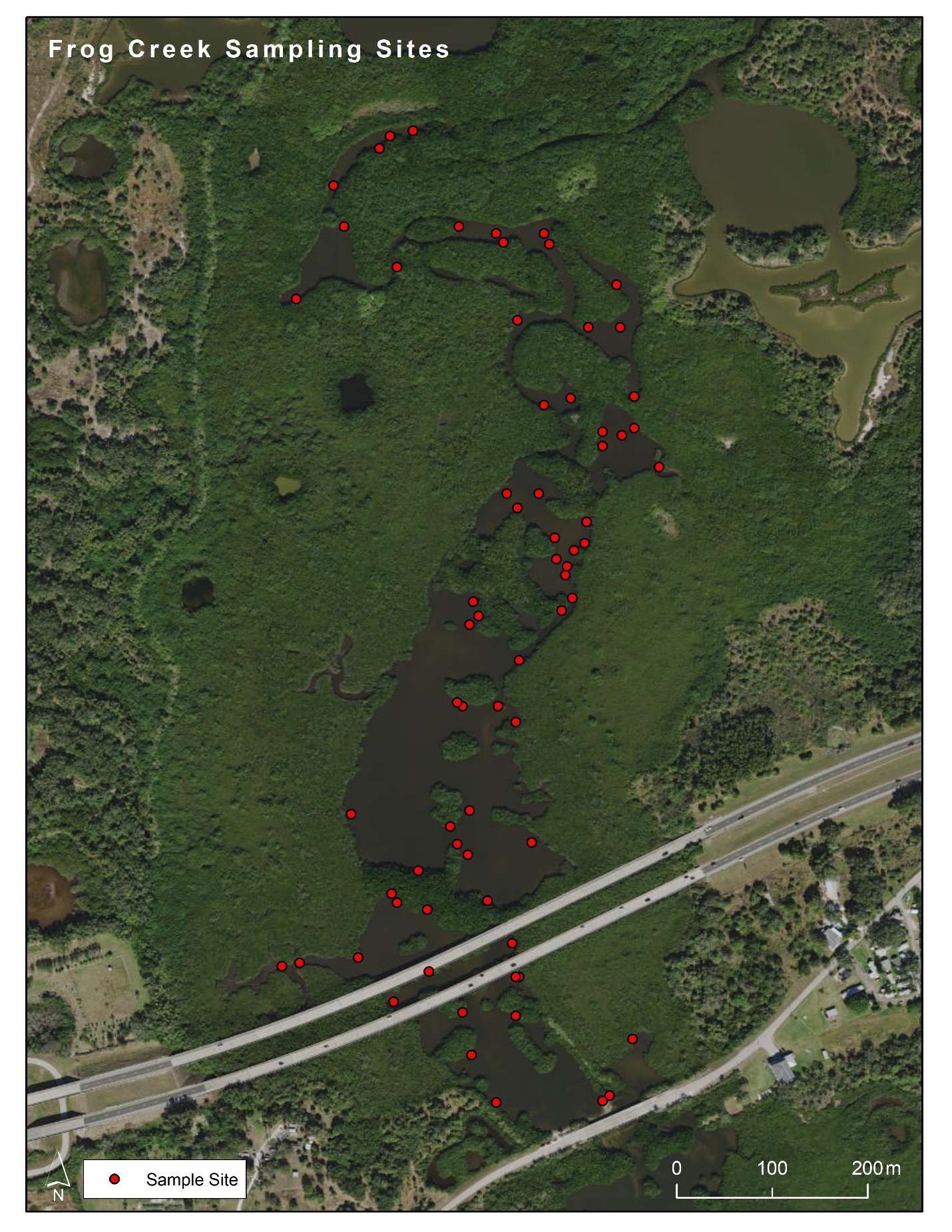


S1C. Natural Site Little Manatee River (LM)


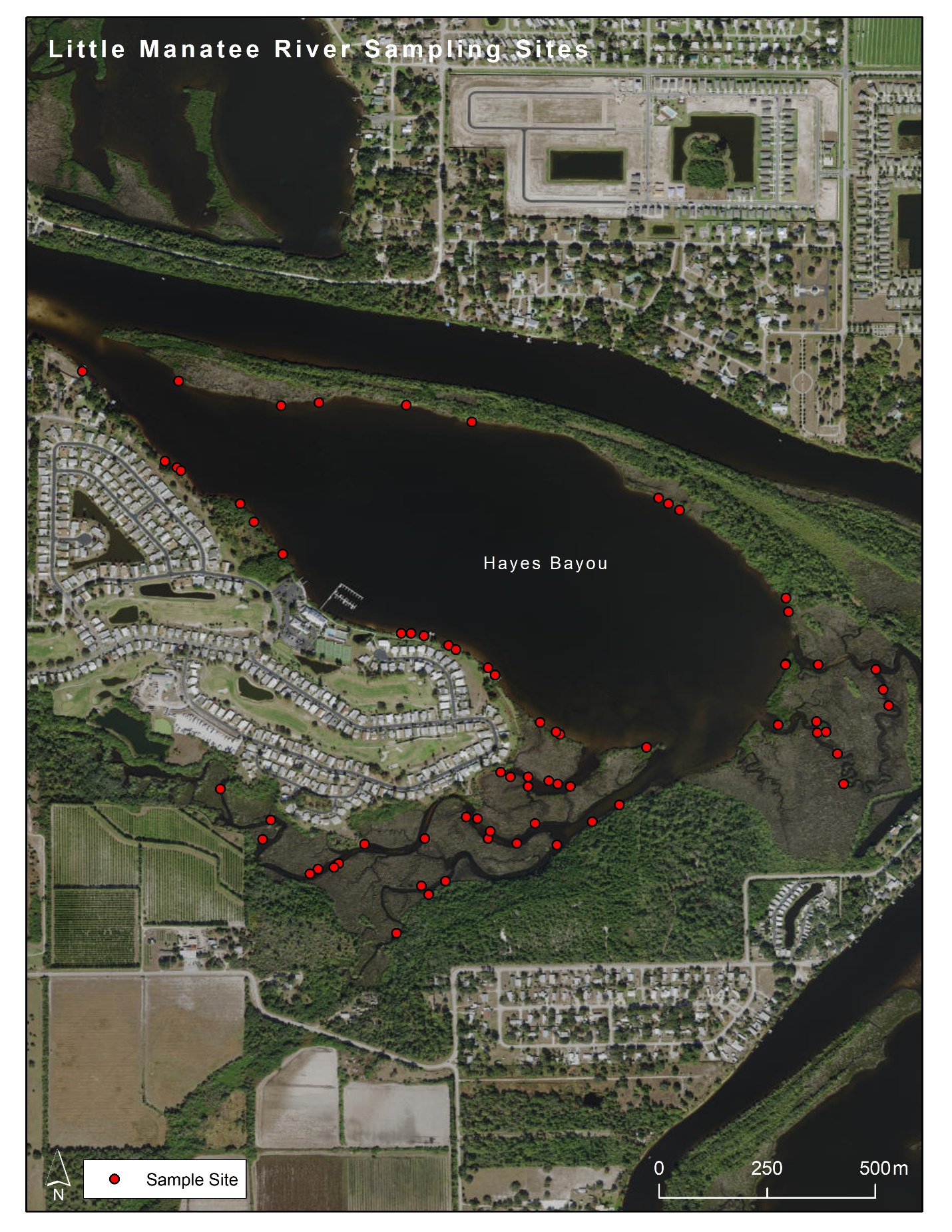


S1D. Restored site Cockroach Bay (CBR)


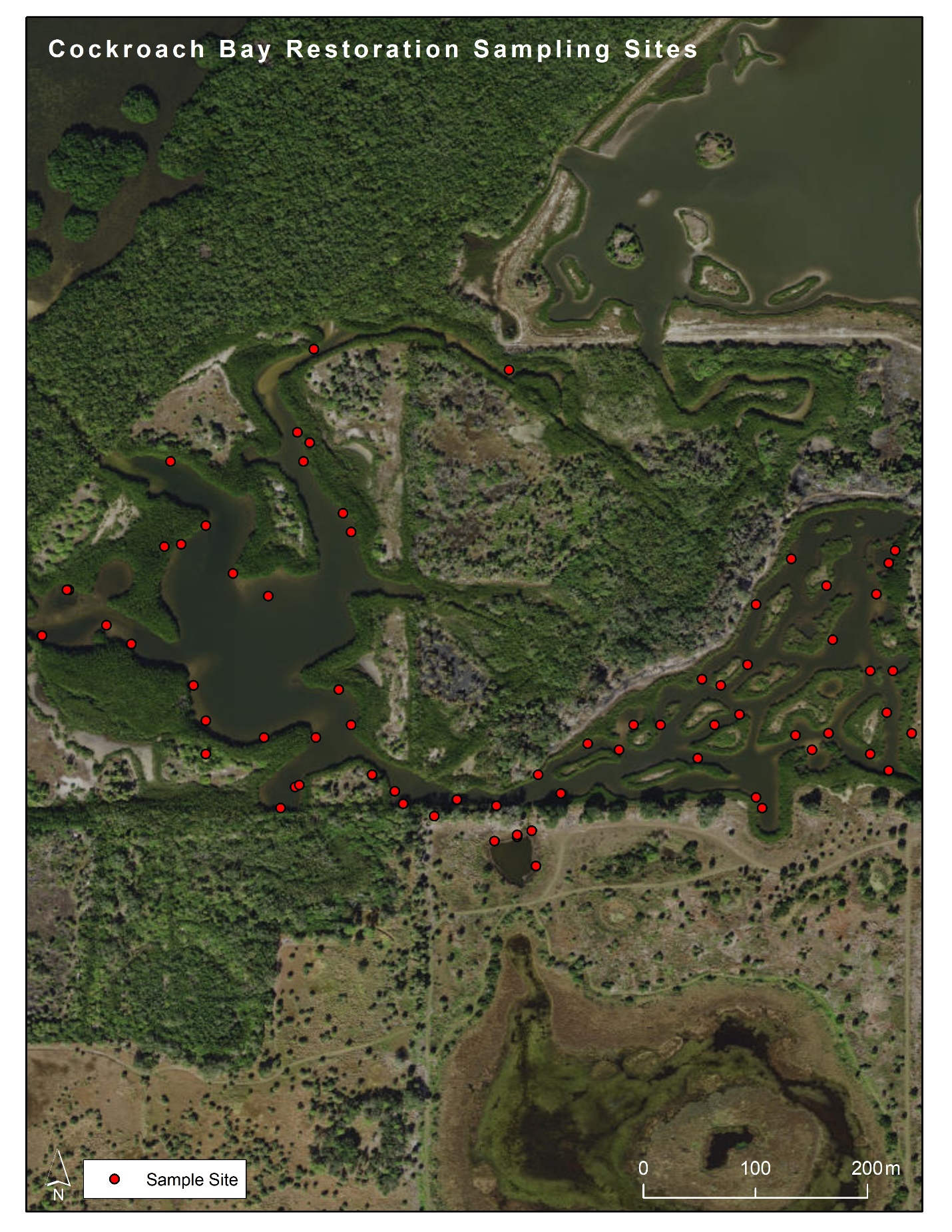


S1E. Restored site Rock Ponds Ecosystem Restoration (RP)


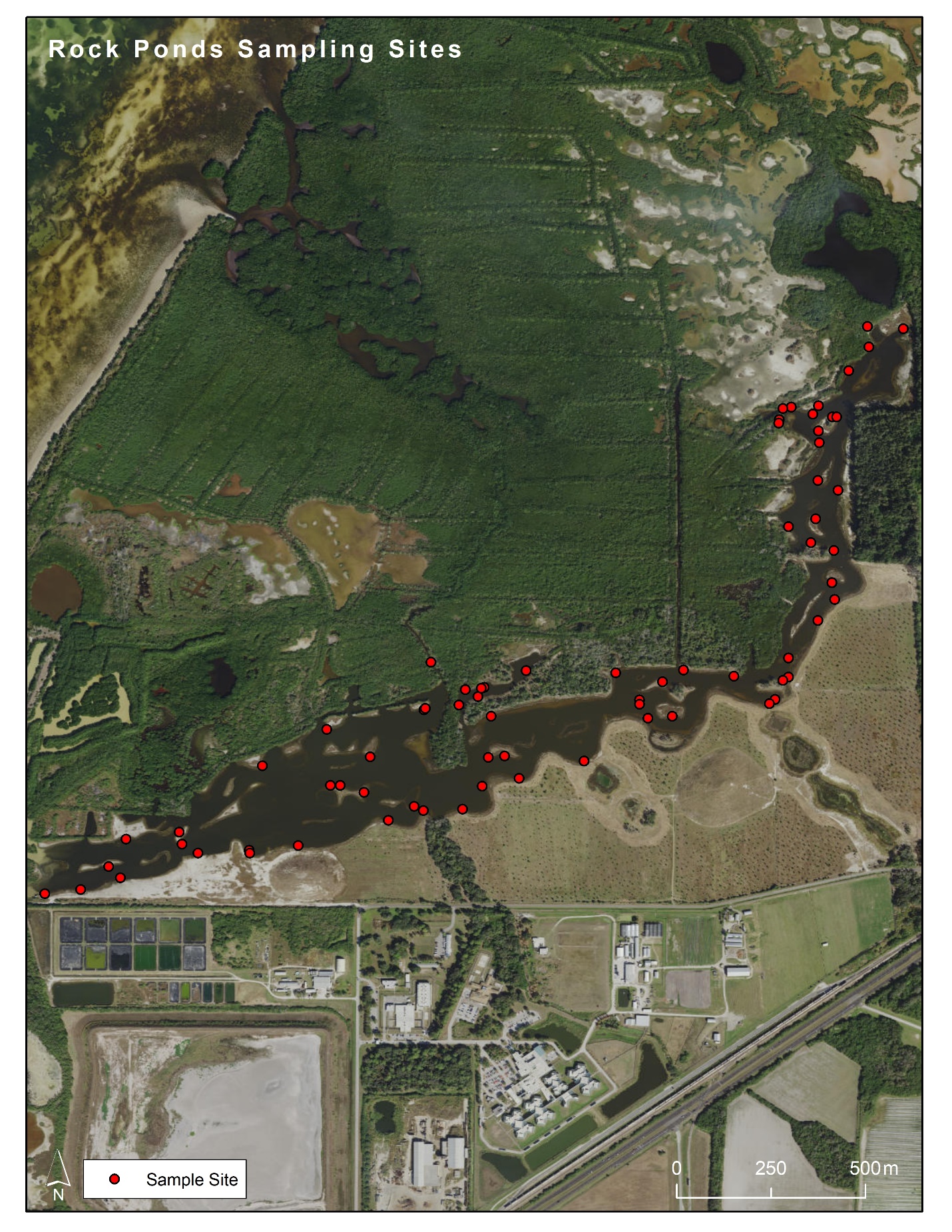


S1F. Restored Site Terra Ceia (TC)


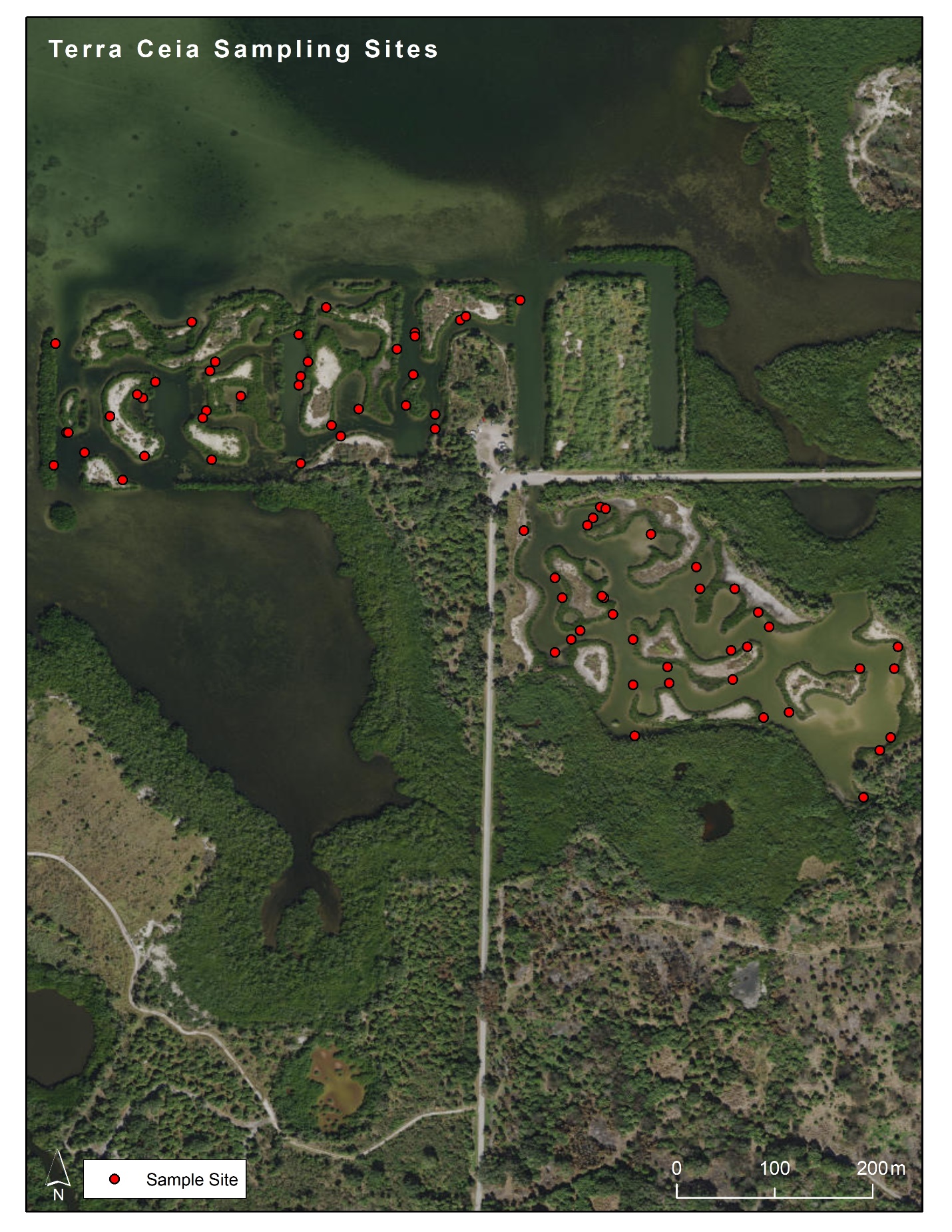


S1G. Impacted site Dug Creek (DC)


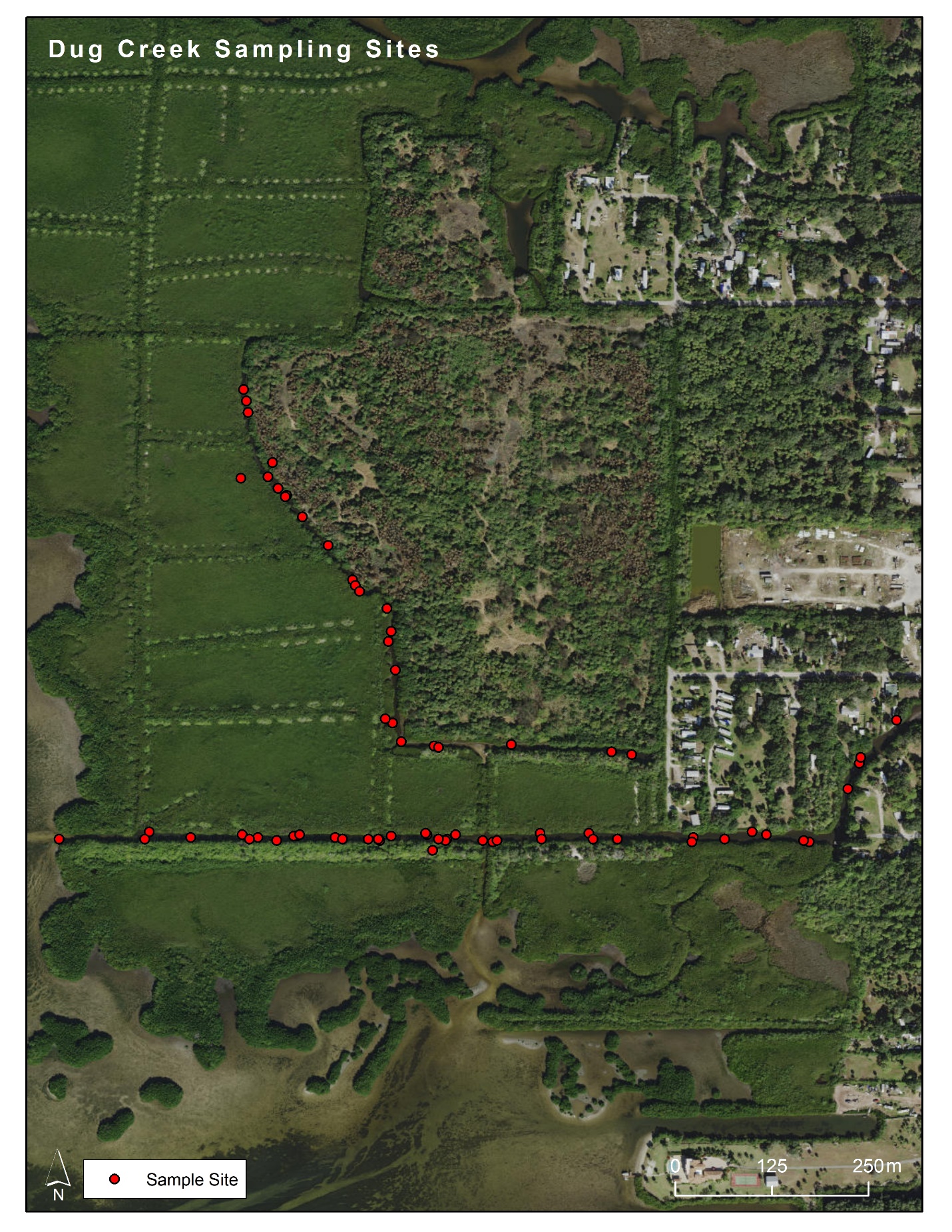


S1H. Impacted site E.G. Simmons (EG)


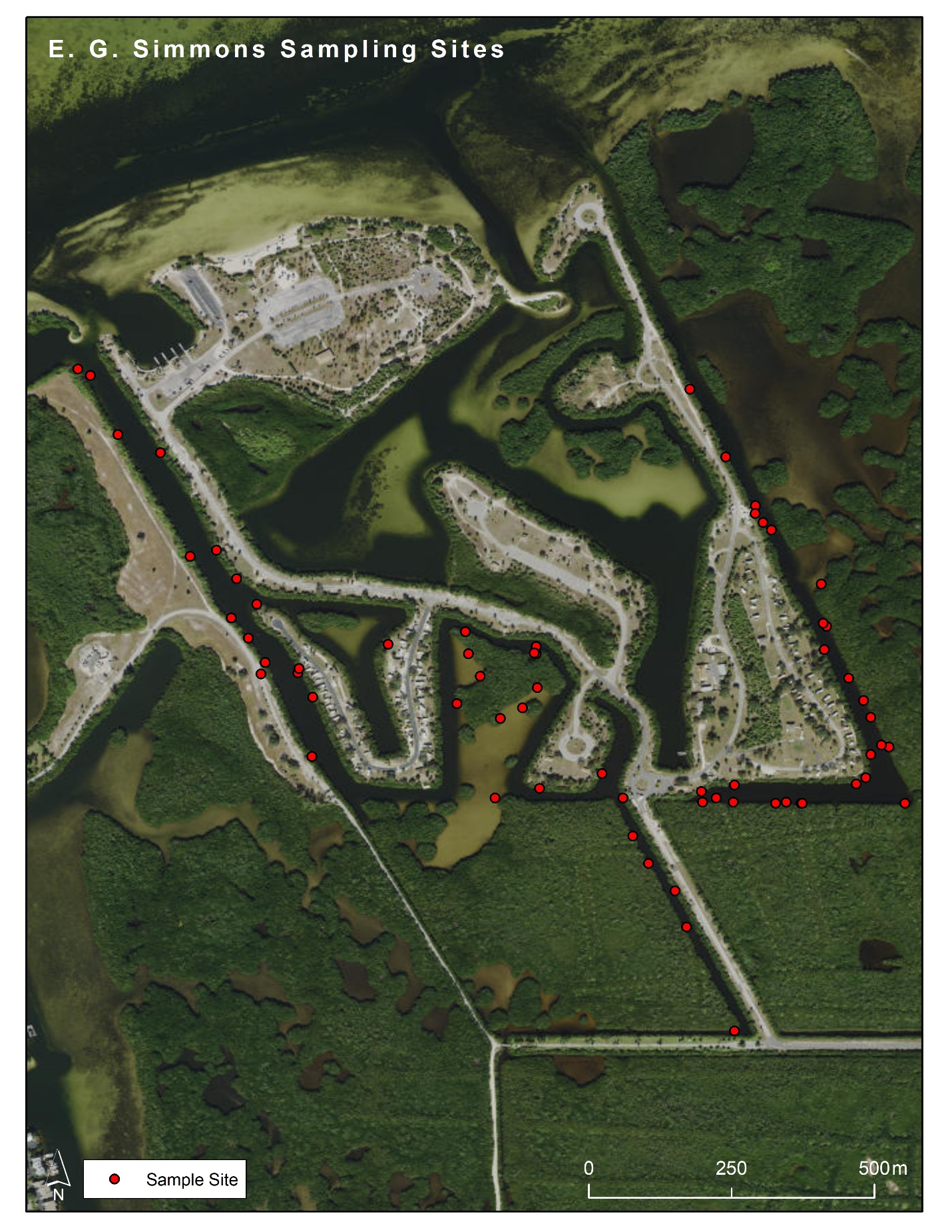


S1I. Impacted site Newman Branch


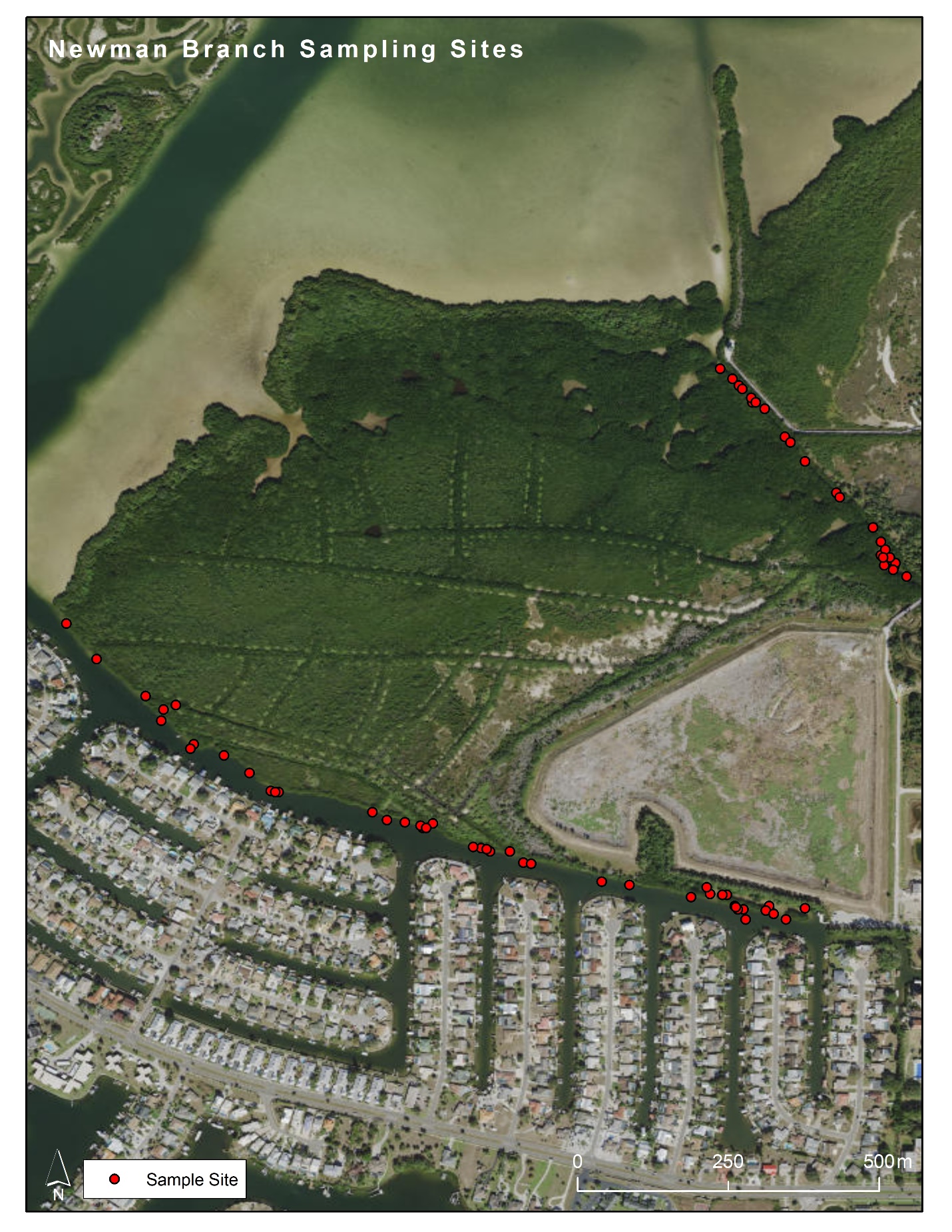

Supplement: S1 Fig — Map images were sources from the State of Florida via ESRI. (DOCX) [file pone.0240623.s001.docx]
