# Supplementary material for: Coastal restoration evaluated using dominant habitat characteristics and associated fish communities
Source: PLoS One. 2020 Oct 22;15(10):e0240623. doi: 10.1371/journal.pone.0240623 (PMC7580894; doi:10.1371/journal.pone.0240623)
Supplement: S1 Table — (DOCX) [file pone.0240623.s002.docx]

**S1 Table. Complete list of fishes captured for both gear types during the entire study.**

| Family | Scientific Name | Common Name | Number in 9-m seine | Number in 40-m seine |
| --- | --- | --- | --- | --- |
| **Achiridae** | *Achirus lineatus* | lined sole | 24 | 6 |
|  | *Trinectes maculatus* | hogchoker | 284 | 69 |
| **Ariidae** | *Ariopsis felis* | hardhead catfish | 9 | 5 |
| **Atherinopsidae** | *Atheriniformes sp.* | silverside sp. | 12,744 | 10 |
| **Batrachoididae** | *Opsanus beta* | gulf toadfish | 16 | 5 |
|  | *Opsanus tau* | oyster toadfish | 1 | 1 |
| **Belonidae** | *Strongylura marina* | Atlantic needlefish | 19 | 9 |
|  | *Strongylura notata* | redfin needlefish | 49 | 29 |
|  | *Strongylura timucu* | timucu | 1 | 0 |
| **Blenniidae** | *Chasmodes saburrae* | Florida blenny | 6 | 0 |
|  | *Hypleurochilus pseudoaequipinnis* | oyster blenny | 1 | 0 |
| **Carangidae** | *Oligoplites saurus* | leatherjacket | 83 | 4 |
| **Centropomidae** | *Centropomus undecimalis* | common snook | 322 | 278 |
| **Cichlidae** | *Cichlasoma urophthalmus* | mayan cichlid | 12 | 1 |
|  | *Oreochromis aureus* | blue tilapia | 1 | 3 |
|  | *Oreochromis niloticus* | nile tilapia | 0 | 1 |
|  | *Sarotherodon melanotheron* | blackchin tilapia | 58 | 53 |
| **Clupeidae** | *Brevoortia patronus* | gulf menhaden | 1 | 0 |
|  | *Dorosoma petenense* | threadfin shad | 88 | 10 |
|  | *Harengula jaguana* | scaled sardine | 3,268 | 143 |
|  | *Opisthonema oglinum* | threadfin herring | 1 | 0 |
| **Cyprinodontidae** | *Cyprinodon variegatus* | sheepshead minnow | 1,402 | 4 |
|  | *Floridichthys carpio* | goldspotted killifish | 14 | 1 |
| **Dasyatidae** | *Dasyatis sabina* | Atlantic stingray | 3 | 7 |
| **Elopidae** | *Elops sarus* | ladyfish | 7 | 17 |
| **Engraulidae** | *Anchoa mitchilli* | bay anchovy | 67,325 | 3 |
| **Ephippidae** | *Chaetodipterus faber* | Atlantic spadefish | 2 | 77 |
| **Fundulidae** | *Fundulus grandis* | gulf killifish | 202 | 5 |
|  | *Fundulus similis* | longnose killifish | 23 | 0 |
|  | *Luciana parva* | rainwater killifish | 2,518 | 24 |
| **Gerreidae** | *Diapterus auratus* | Irish pompano | 77 | 47 |
|  | *Eucinostomus gula* | silver jenny | 260 | 154 |
|  | *Eucinostomus Havana* | bigeye mojarra | 16 | 0 |
|  | *Eucinostomus harengulus* | tidewater mojarra | 2,293 | 565 |
|  | *Eugerres plumieri* | striped mojarra | 161 | 85 |
|  | Gerreidae sp. | mojarra sp. | 2,026 | 6 |
| **Gobiidae** | *Bathygobius soporator* | frillfin goby | 3 | 0 |
|  | *Ctenogobius boleosoma* | darter goby | 1 | 0 |
|  | *Gobionellus oceanicus* | highfin goby | 8 | 2 |
|  | *Gobiosoma bosc* | naked goby | 51 | 0 |
|  | *Gobiosoma robusum* | code goby | 84 | 0 |
|  | *Lophogobius cyprinoides* | crested goby | 283 | 4 |
|  | *Microgobius gulosus* | clown goby | 1,262 | 1 |
| **Haemulidae** | *Orthopristis chrysoptera* | pigfish | 1 | 0 |
| **Lepisosteidae** | *Lepisosteus osseus* | longnose gar | 0 | 1 |
|  | *Lepisosteus platyrincus* | Florida gar | 1 | 0 |
| **Lutjanidae** | *Lutjanus griseus* | gray snapper | 11 | 5 |
| **Megalopidae** | *Megalops atlanticus* | tarpon | 0 | 3 |
| **Muglidae** | *Mugil cephalus* | striped mullet | 441 | 45 |
|  | *Mugil curema* | white mullet | 116 | 5 |
|  | *Mugil gyrans* | whirligig mullet | 116 | 5 |
| **Paralichthyidae** | *Paralichthys albiguttata* | gulf flounder | 1 | 2 |
| **Poecilidae** | *Belonesox belizanus* | pike killifish | 66 | 4 |
|  | *Gambusia holbrooki* | eastern mosquitofish | 201 | 0 |
|  | *Poecilia latipinna* | sailfin molly | 252 | 6 |
| **Rhinopteridae** | *Rhinoptera bonasus* | cownose ray | 0 | 3 |
| **Sciaenidae** | *Bairdella chrysoura* | silver perch | 47 | 2 |
|  | *Cynoscion nebulosus* | spotted seatrout | 47 | 0 |
|  | *Leiostomus xanthurus* | spot | 1,005 | 189 |
|  | *Menticirrhus americanus* | southern kingfish | 1 | 0 |
|  | *Micropogonias undulatus* | Atlantic croaker | 0 | 1 |
|  | *Pogonias cromis* | black drum | 115 | 40 |
|  | *Sciaenops ocellatus* | red drum | 494 | 25 |
| **Sparidae** | *Archosargus probatocephalus* | sheepshead | 99 | 27 |
|  | *Lagodon rhomboides* | pinfish | 1,716 | 208 |
| **Sphyraenidae** | *Sphyraena barracuda* | great barracuda | 0 | 1 |
| **Syngnathidae** | *Hippocampus zosterae* | dwarf seahorse | 4 | 0 |
|  | *Syngnathus floridae* | dusky pipefish | 1 | 0 |
|  | *Syngnathus scovelli* | gulf pipefish | 72 | 4 |
| **Synodontidae** | *Synodus foetens* | inshore lizardfish | 10 | 0 |
| **Tetraodontidae** | *Sphoeroides nephelus* | southern pufferfish | 9 | 0 |
| **Triglidae** | *Prionotus tribulus* | bighead searobin | 1 | 0 |
