# Supplementary material for: Coastal restoration evaluated using dominant habitat characteristics and associated fish communities
Source: PLoS One. 2020 Oct 22;15(10):e0240623. doi: 10.1371/journal.pone.0240623 (PMC7580894; doi:10.1371/journal.pone.0240623)
Supplement: S2 Table — (DOCX) [file pone.0240623.s003.docx]

**S2 Table. A list of the commercially and/or recreationally important sportfish captured during the study.**

| Family | Scientific Name | Common Name | Number in 9-m seine | Number in 40-m seine |
| --- | --- | --- | --- | --- |
| **Centropomidae** | *Centropomus undecimalis* | common snook | 322 | 278 |
| **Elopidae** | *Elops sarus* | ladyfish | 7 | 17 |
| **Lutjanidae** | *Lutjanus griseus* | gray snapper | 11 | 5 |
| **Megalopidae** | *Megalops atlanticus* | Atlantic tarpon | 0 | 3 |
| **Muglidae** | *Mugil cephalus* | Striped mullet | 441 | 45 |
|  | *Mugil curema* | White mullet | 116 | 5 |
|  | *Mugil trichodon* | fantail mullet | 104 | 2 |
| **Paralichthyidae** | *Paralichthys albiguttata* | gulf flounder | 1 | 2 |
| **Sciaenidae** | *Cynoscion nebulosus* | spotted seatrout | 47 | 0 |
|  | *Menticirrhus americanus* | southern kingfish | 1 | 0 |
|  | *Micropogonias undulates* | Atlantic croaker | 0 | 1 |
|  | *Pogonias cromis* | black drum | 115 | 40 |
|  | *Sciaenops ocellatus* | red drum | 494 | 25 |
|  | *Archosargus probatocephalus* | sheepshead | 99 | 27 |
|  | *Sphyraena barracuda* | great barracuda | 0 | 1 |
